# Increased Synapse Formation Obtained by T Cell Epitopes Containing a CxxC Motif in Flanking Residues Convert CD4+ T Cells into Cytolytic Effectors

**DOI:** 10.1371/journal.pone.0045366

**Published:** 2012-10-09

**Authors:** Vincent A. Carlier, Luc VanderElst, Wim Janssens, Marc G. Jacquemin, Jean-Marie R. Saint-Remy

**Affiliations:** Center for Molecular and Vascular Biology, University of Leuven, Leuven, Belgium; Tulane University, United States of America

## Abstract

The nature of MHC class II-binding epitopes not only determines the specificity of T cell responses, but may also alter effector cell functions. Cytolytic CD4+ T cells have been observed primarily in anti-viral responses, but very little is known about the conditions under which they can be elicited. Their potential as regulators of immune responses, however, deserves investigations. We describe here that inclusion of a thiol-disulfide oxidoreductase motif within flanking residues of class II-restricted epitopes results, both in vitro and in vivo, in elicitation of antigen-specific cytolytic CD4+ T cells through increased synapse formation. We show that both naïve and polarized CD4+ T cells, including Th17 cells, can be converted by cognate recognition of such modified epitopes. Cytolytic CD4+ T cells induce apoptosis on APCs by Fas-FasL interaction. These findings potentially open the way towards a novel form of antigen-specific immunosuppression.

## Introduction

Naïve CD4+ T cells acquire various phenotypes during peripheral activation and expansion. Acquisition of a phenotype depends on many factors, including the nature of the antigen-presenting cell, the location at which such activation occurs, the presence of soluble factors including cytokines, co-stimulatory molecules, autocrine and paracrine signals from T cells themselves [1].

Polarized CD4+ T cells, on the other hand, have until recently been considered as terminally differentiated, with no or only limited plasticity. However, this view was recently challenged based on observations showing that IL-17 producing cells can be converted to regulatory T cells and vice versa [2], [3]. Analysis of gene expression has in parallel demonstrated that even polarized CD4+ T cells retain bivalent markers at transcription factor genes, predicting at least some degree of plasticity within the polarized CD4+ T cell repertoire [4].

Recent evidence has suggested that T cell stimulation strength could be instrumental in dictating the fate of T cells. Such strength represents the sum of signals provided by antigen affinity and density, amplification depending of costimulatory signals and duration of the synapse formed with antigen-presenting cells [5]. One illustration of this has been provided by the demonstration that low-strength activation was required to promote a Th17 cell phenotype [6].

MHC class II molecules can accommodate epitopes of up to 20 aminoacids from which 9 constitute the core sequence inserted into class II cleft [7].

We have investigated the possibility of varying amino acid residues located in epitope flanking regions to modulate the strength of the synapse formed by cognate recognition of a peptide-MHC complex, thereby altering CD4+ T cell properties.

These studies were encouraged by our previously reported observations [8], in which a CD4+ T cell clone acquired cytolytic properties with induction of apoptosis of antigen-presenting cells by exposure to an epitope containing a cysteine in its flanking residues.

Cytolytic CD4 (cCD4) T cells have been described on occasions over the last 20 years, associated with immune responses to viruses [9], both during natural disease [10], [11] and as an outcome of vaccination [12]. Their importance in tumor elimination seems to have been underestimated [13]. Yet, the conditions under which they can be elicited, either in vitro or in vivo, have not been explored in details, despite potential therapeutic usefulness.

The studies reported here now provide the demonstration that activation of CD4+ T cells by natural peptides encompassing class II-restricted epitopes and a thiol-disulfide oxidoreductase motif within flanking residues is sufficient to increase the strength of CD4 T cell stimulation. This results in acquisition of cytolytic properties and elimination of APCs by apoptosis induction.

## Results

### The cytolytic properties of murine CD4+ T cell clones to p21–35 depend on the presence of a thiol-disulfide oxidoreductase motif within epitope flanking residues

One possibility to increase synapse strength is to introduce cysteine residues within epitope flanking regions. We previously reported on a murine CD4^+^ T cell clone (G121) with a CD25^hi^CD28^−^ phenotype at rest, which induced apoptosis of WEHI-231 cells upon cognate recognition of a class II-restricted peptide, p21–35 [8]. These unexpected properties prompted us to further characterize the conditions under which such T cell clones could be obtained.

The initial G121 clone was induced by immunization of BALB/c mice (H-2^d^) with p21–35 in CFA/IFA and required several cycles of stimulation in vitro for full expression of cytolytic properties. To first exclude that the adjuvant determined the induction of cytolytic properties, we immunized BALB/c mice with the peptide adsorbed on alum, which elicited CD4+ T cells with comparable cytolytic activity. Interestingly, however, this activity was observed ex vivo with no requirement for further in vitro stimulation (data not shown).

Epitope 21–35 (CHGSEPCIIHRGKPF) has been extensively characterized in our laboratory. It contains a promiscuous T cell epitope of the Der p 2 allergen restricted by class II determinants, H-2A^d^ or H-2A^b^ [14], [15]. MHC class II anchoring residues were identified, with residue E25 in P(1), leaving four aminoterminal flanking residues.

Flanking residues of class II peptides can stabilize peptide-MHC (pMHC) conformers by interfering with H2-DM editing activity [16]. Although the presence of a serine in 21–35 P(−1) rendered it unlikely, we considered the possibility that induction of cytolytic properties could result from such stabilization. Stabilized pMHC conformers are recognized by peptide-specific T cell clones but not by T cells elicited towards the whole corresponding antigen [16]. We therefore immunized mice with full-length Der p 2 in alum and we obtained several cytolytic CD4+ T cell clones that were activated by both p21–35 and antigen (data not shown).

**Figure 1:**
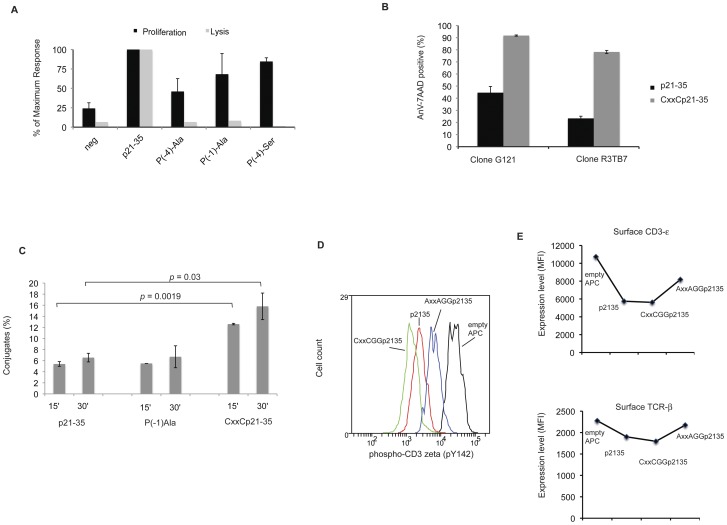
Effects of aminoacid residue mutation in the p21–35 flanking sequence. (A) Proliferation response and cytolytic properties of a p21–35 specific CD4+ T cell clone (G121) obtained from peptide-immunized BALB/c mice. Various mutant peptides (0.1 µM; sequence as indicated) were cultured with mitomycin C-treated splenocytes (T cell depleted). After 48 h, ^3^H-thymidine was added and its incorporation measured after 18 h of culture. Results are shown as percentage of incorporation obtained by comparison with p21–35 wildtype sequence (containing a CxxS motif). G121-induced cytolysis was measured on Dioc18-stained WEHI 231 B cells loaded with 0.1 µM of each peptide. After 24 h of incubation, cells were stained with Annexin V and 7-AAD and analysed by flow-cytometry. WEHI cell lysis (Dioc18+) is represented as percentage of lysis obtained by comparison with p21–35 wildtype sequence. Error bars represent 1 standard deviation (SD). Data are representative of three independent experiments. (B) Cytolytic assay of WEHI 231 B cells carried out as in (A) using two different CD4+ clones (G121 and R3TB7) exposed to peptides in either p21–35 wildtype sequence or after introduction of a cysteine in P(−1) thereby creating a thio-oxidoreductase motif. Error bars represent 1 SD. Data representative of two experiments. (C) Conjugate formation. DioC18 stained WEHI 231 B cells were loaded for 1 h with 1 µM of either Der p 2 p21–35, mutant peptide P(−1)Ala or with peptide containing the sequence CxxCGG. A CellTrace DDAO stained p21–35 specific clone (R3TB7) was added to each WEHI cell culture (ratio 1/1) and cell mixtures were briefly centrifuged. After 15 or 30 minutes, cells were softly resuspended, fixed for 10 minutes with 4% paraformaldhyde and analyzed by flow cytometry. Percentages of conjugates stand for the proportion of T cells staining positive for both labels over the total T cell population. Error bars represent 1 SD. Two tailed *p* values are derived from unpaired t tests. Data representative of two experiments. (D) Catabolism of CD3-ζ. On day 2 after restimulation, a p21–35 specific clone R3TB6 was washed and cultured for 15 minutes with WEHI 231 B cells either unloaded or preloaded with p21–35, CxxCGGp21–35 or loss of function peptide AxxAGGp21–35 (50 µM each). After fixation and permeation, expression of phosphorylated CD3-ζ chain was determined by flow cytometry. Data representative of three experiments and different p21–35 specific cell lines. (E) The same cells as in (D) were stained for surface CD3-ε or TCR-β after 1 h of culture with WEHI 231 B cells either unloaded or preloaded with p21–35, CxxCGGp21–35 or loss of function peptide AxxAGGp21–35 (50 µM each). Data representative of two experiments.

The CHGS sequence of the aminoterminal flanking residues of p21–35 contains a thiol-oxidoreductase motif (CxxS; thioreductase in short), characteristic of monocysteinic glutaredoxins [17]. We pondered whether this motif was essential in determining the properties of CD4+ T cells. To this end, P(−1) or P(−4) residues of the flanking sequence were replaced with alanine, or by alanine or serine, respectively. Figure 1A shows that each of these three substitutions resulted in a complete abolition of cytolytic activity of the CD4+ T cell clone tested. Replacement of both residues by alanine had the same result (data not shown). This demonstrated that the cytolytic activity was dependent on an intact CxxS motif.

Proliferation was reduced by 50% with alanine in P(−4) but not when serine was used, and was only marginally affected when P(−1) serine was substituted by alanine.

Therefore, a cysteine or serine in P(−4) was required for optimal proliferation, whilst substitution in P(−1) has little effect. Previous experiments had shown that aminoacids in P(−4) could not interact with MHC determinants [18]. It was therefore hypothesized that cysteine or serine in P(−4) interacted with components of the synapse at T cell level, possibly by increasing the contact time between cells, whilst acquisition of cytolytic properties required a full CxxC or CxxS motif (see below).

Notably, proliferation required the integrity of the peptide sequence included in the class II MHC cleft. We have reported that substitution of P(4) isoleucine by asparagine significantly reduced the proliferation of p21–35-specific T cell clones by reducing the affinity for MHC class II determinants [14].

We reasoned that if a thioreductase activity was required, then substitution of serine in P(−1) with a cysteine, thereby forming a thioredoxin-like motif (CxxC), could enhance the capacity of the peptide to elicit cytolytic CD4+ T cells. The oxido-redox potential of thioredoxin is significantly higher than that of glutaredoxin [19]. This prediction was correct: the CxxC motif increased the cytolytic properties (Figure 1B) without alteration of T cell proliferation (Figure S1). We then verified that peptides containing an oxido-redox motif efficiently reduced disulfide bridges using a fluorescence-based reduction assay [20], in which we further observed that peptides containing a CxxC motif were more efficient than those with a CxxS motif (Figure S2).

Inserting a CxxC motif within the flanking residues of class II-restricted peptides could provide a strategy by which the properties of alternative T cell epitopes could be altered. To prevent interactions between TCR and proximal flanking residues [16], we introduced two glycine residues between the motif and the P(1) residue. This did not alter peptide capacity to induce T cell proliferation (Figure S1). We therefore decided to introduce a linker made of two glycines in all peptides used from then on (see below).

The link between the presence of an active CxxC motif and its consequences on T cell properties suggested the existence of a target protein at the surface of CD4+ T cells or APCs. Incubation of effector CD4+ T cell clones with soluble peptides containing a CxxC motif did not elicit cytolytic properties, nor when T cells were incubated with free thioredoxin (data not shown). This suggested that the thioreductase activity interfered with a T cell surface molecule participating in immune synapse (IS) formation. From previous experiments [14], we knew that p21–35 required processing for efficient MHC class II-restricted presentation. We therefore incubated T cell-depleted splenocytes with different forms of p21–35 and we measured the kinetics of dimer formation between the APC and a specific T cell clone. Figure 1C shows that the formation of conjugates was significantly increased (up to 3-fold) when peptides containing a CxxC motif were used.

Increased cell proliferation can be due to inability to organize the cSMAC (an activation cluster at the center of the immune synapse), even when conjugates and synapse have formed [21]. In CD4+ T cells, TCR degration is known to occur mainly at the cSMAC when B cells are used as APC [5], [22]; we therefore used this parameter as a marker of mature immune synapse formation [21]. Figure 1D shows the level of phosphorylated CD3-ζ chain of a cycling p21–35 specific T cell clone (day 2) upon stimulation with WEHI 231 B cells loaded with different forms of peptides (after repeating stimulations with no resting period, such population show sustained phosphorylation of CD3-ζ, making such T cell population a sensitive tool to detect CD3-ζ degradation; Carlier *et al* unpublished observations). The highest TCR degradation was observed with epitopes containing the CxxC motif as compared to a loss-of-function epitope with an AxxA motif, with intermediate results obtained with the natural epitope (containing a CxxS motif). In Figure 1E, degradation of TCR was confirmed by analysing surface CD3-ε and TCR-β expression. A direct correlation was therefore established in between the redox potential of epitopes and capacity to increase the strength of TCR degradation.

The possibility still existed that p21–35 specific CD4+ T cell clones acquired a cytolytic phenotype because of clonotypic selection. We sequenced a number of cytolytic CD4+ T cells (cCD4) obtained from different immunizations with p21–35. The majority of the clones were Vβ-8(+) but various alternative beta chains were used. Sequencing of the beta chain CDR3 of four of Vβ-8(+) clones identified significant variations between clones, ruling out the hypothesis that cytolytic properties were confined to a specific T cell clonotype (data not shown).

Taken together, these data show that a class II-restricted T cell epitope including a CxxC motif within its flanking residues have the capacity to elicit cytolytic CD4+ T cells, seemingly by increasing the strength of the IS. This phenotypic transformation of CD4+ T cells occured without altering the sequence of aminoacids directly recognized by TCR, thereby standing in contrast with altered peptide ligands, in which either MHC anchoring- or T cell contact residues are modified [23]. cCD4+ T cells, once elicited, should recognize natural T cell epitopes, a pre-requisite for establishing physiological relevance.

### Addition of a CxxC motif increases agonistic properties of class II restricted T cell epitopes on naïve CD4+ T cells

We went on determining whether findings obtained with a single T cell epitope could be reproduced with alternative class II-restricted peptides. Data included in Figure 1A show that a significant decrease in proliferation resulted from aminoacid substitution leading to loss of the oxidoreductase motif. We took advantage of this property and compared CD4+ T cells activated by either natural epitopes or their modified counterpart containing a CxxC motif.

We selected three extensively studied class–II restricted T cell epitopes for which contact residues for MHC class II and TCR binding were known and for which transgenic TCR cells were available: the myelin oligodendrocytic glycoprotein (MOG) peptide 35–55 [24], the Dby peptide 605–619 [25] and the Ova peptide 323–339 [26]. The corresponding TCR transgenic mice were in the C57BL/6 background (2D2 for the MOG peptide and Dby for Marilyn mice) or in the BALB/c background for DO11.10, Ova specific peptide. Naïve CD4+CD62L+ TCR transgenic CD4+ T cells were prepared from the spleen of animals of each strain.

**Figure 2:**
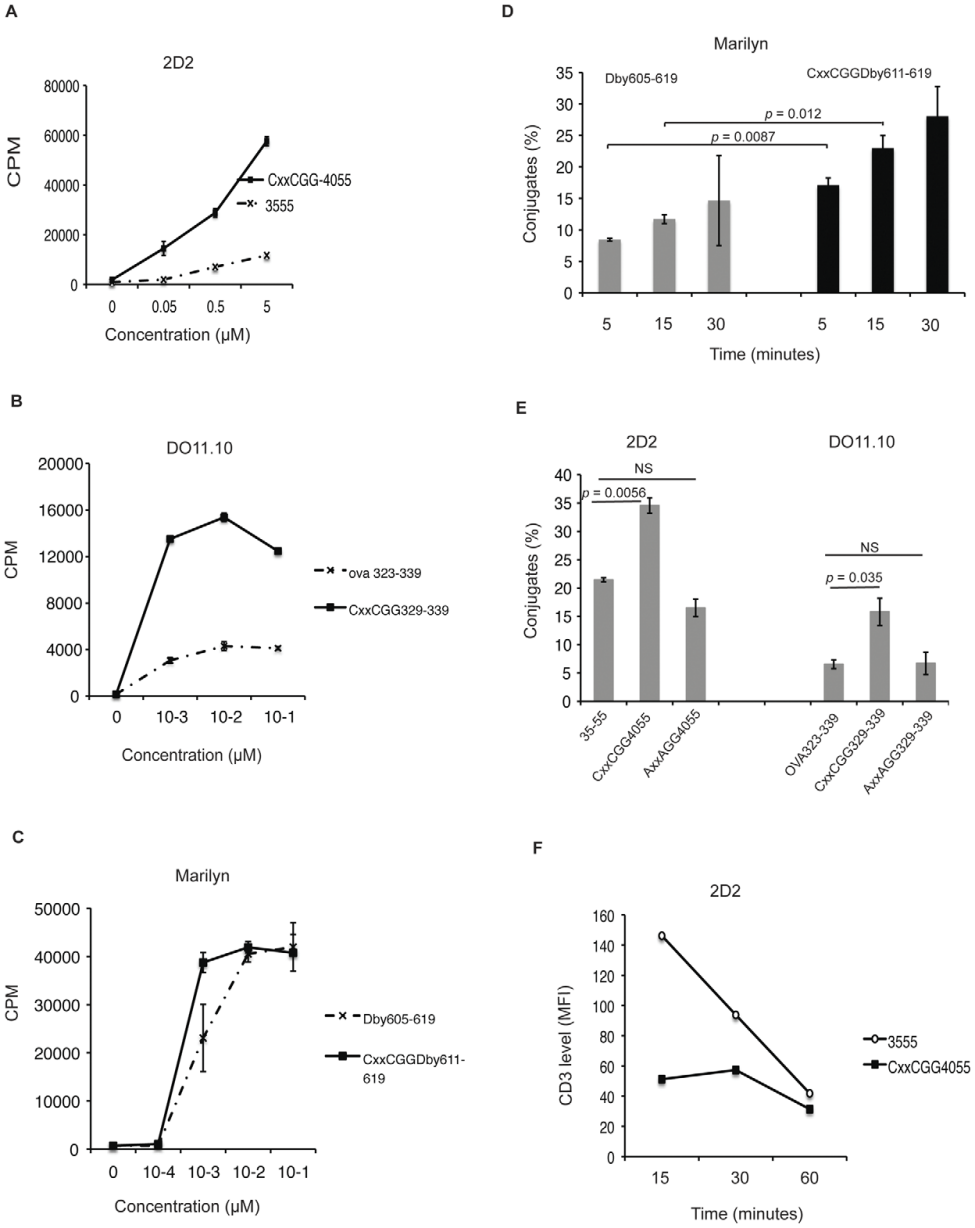
CD4+ T cell stimulatory capacity of modified peptides. CD4+CD62L+ splenic T cells (naïve T cells) were purified from 2D2 TCR transgenic mice (A), DO11.10 transgenic mice (B) or Marylin TCR transgenic mice (C). (A,B,C) The proliferative response of naive cells was assayed after 72 h in cocultures with mitomycin C-treated T cell-depleted splenocytes loaded with indicated peptide concentrations. ^3^H-thymidine was added for the last 12 h of culture. Error bars represent 1 SD. Data representative of three experiments. (D) Conjugate formation. C57BL/6 T cell-depleted splenocytes were stained with DIOC18, preloaded with 1 µM of either natural sequence or peptide modified as indicated, and cultured with CellTrace DDAO-SE far red stained CD4+CD62L+ T cells from Marylin transgenic mice (1/1 ratio). At indicated time-points, cells were gently resuspended, fixed for 10 minutes and analysed by flow cytometry. The percentage of cells forming conjugates was calculated as described in Figure 1C. Error bars represent 1 SD. Two tailed *p* values are derived from unpaired t tests. Data representative of two experiments. (E) Same experiment as in (D), with CD4+CD62L+ T cells from either 2D2 TCR or DO11.10 mice. Conjugates formation was analysed after 30 minutes of culture. Data representative of two experiments. Two tailed *p* values are derived from unpaired t tests. Error bars represent 1 SD. (F) Kinetics of CD3 or TCR downregulation. CD4+CD62L+ T cells purified from 2D2 transgenic mice were stimulated with T cell depleted splenocytes loaded with 50 µM natural sequence or modified peptide containing a CxxC motif. At indicated time points, cells were resuspended, stained with anti-CD4 and anti-CD3ε antibodies followed by analysis by flow-cytometry. Data set is representative of two experiments.

The stimulatory capacity of each pair of peptides (with or without a CxxC motif) was compared in proliferation assays using T cell depleted splenocytes as APC. Figures 2A, B and C show that in each case a significant increase in proliferation was obtained with peptides containing the thioreductase motif.

We next examined whether increased proliferation correlated with increased conjugate formation [21], [22]. Similar results were obtained with each of the three transgenic T cells. Data are illustrated in Figure 2D for the Dby-specific T cells with increased conjugate formation at all time points, and for MOG- and OVA-specific transgenic T cells at 30 minutes (Figure 2E). In each case, the peptide containing the CxxC motif showed increased conjugate formation. Substitution of cysteines in the motif by alanines reduced the extent of conjugate formation to that obtained with the natural epitope.

The strength at which the synapse is formed correlates with increased TCR downregulation [22]. Using an anti-CD3 antibody we followed surface expression of CD3 over time. Results are shown for the MOG transgenic T cells (Figure 2F), but similar results were obtained for the three clones, confirming fast downregulation of CD3 when the peptide contained the CxxC motif. All results concurred in demonstrating that peptides containing the CxxC motif showed increased agonistic properties, leading to fast naïve T cell activation, increased formation of cell conjugates and increased early signaling through the TCR.

### Polarized CD4+ T cells respond to peptides containing a CxxC motif by increased proliferation and enhanced synapse formation

Thus far, we have demonstrated that naïve CD4+ T cells responded to CxxC motif-containing peptides by increased proliferation and synapse formation. The plasticity of naïve CD4+ T cells is, however, higher than that of effector cells [27]. It was therefore of interest to examine whether polarized CD4+ T cells were also responsive. The results presented above with p21–35 specific clones (Figure 1A–D) were not considered as conclusive, as the natural p21–35 peptide contained a monocysteinic glutaredoxin motif.

**Figure 3:**
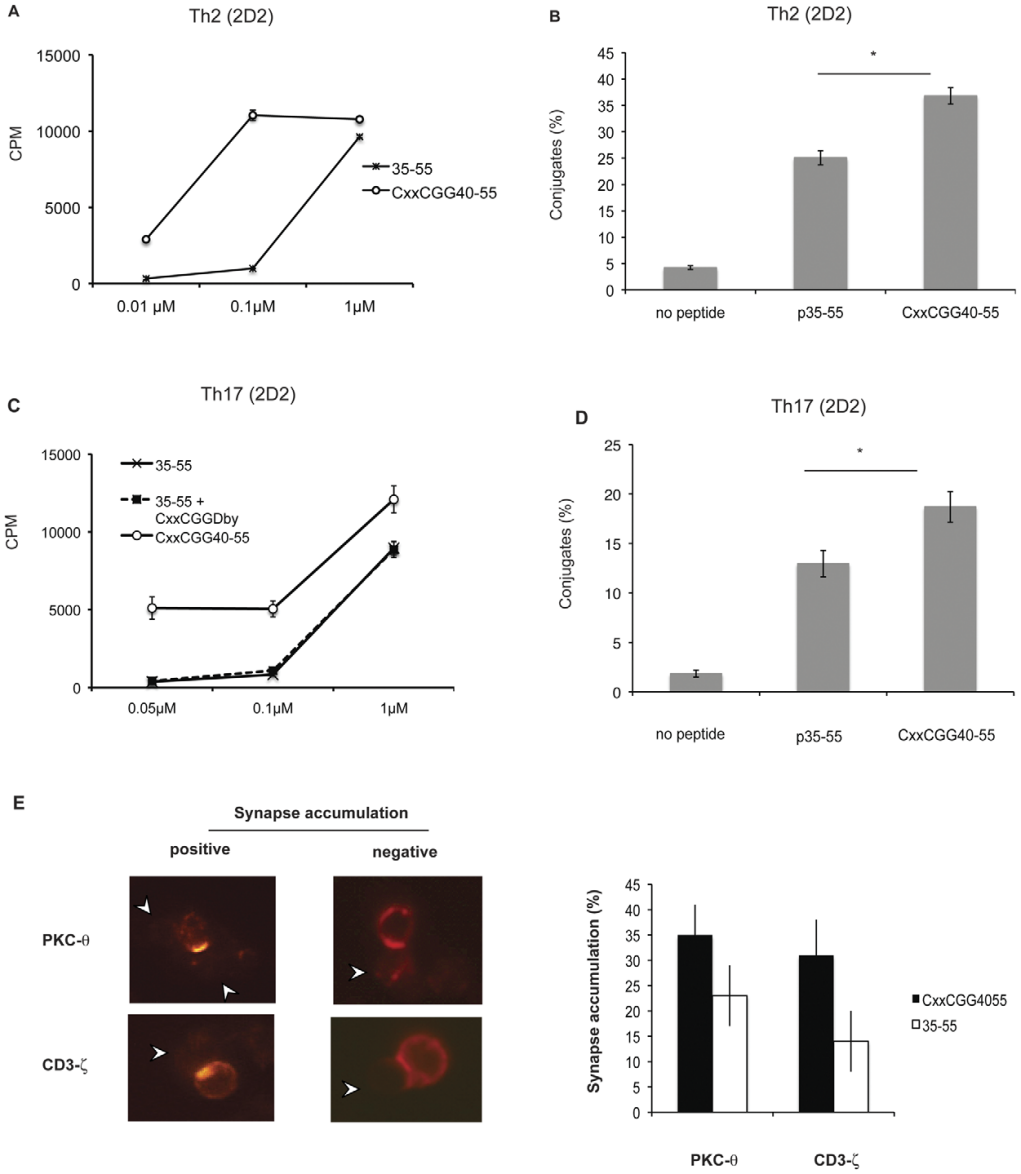
Polarized CD4+ T cells show significantly higher proliferation and conjugate formation when stimulated with peptides containing a CxxC motif. (A) CD4+CD62L+ T cells obtained from 2D2 transgenic mice were polarized by 3 cycles of stimulation under Th2 conditions by addition of IL-4, anti-IFN-γ and anti-IL-12 antibodies in the presence of 1 µM MOG35–55. On day 14 after the last stimulation, cells were cultured for 72 h with mitomycin C-treated T cell-depleted splenocytes from naive C56BL/6 mice in the presence of indicated concentrations of either MOG35–55 or MOG CxxCGG40–55. ^3^H-thymidine was added for the final 12 h culture. Error bars represent 1 SD. Data are representative of two experiments. (B) Conjugate formation. CD4+ T cells from 2D2 transgenic mice were polarized under Th2 conditions and MOG35–55 as described in (A). Cells were then cultured with DiOC18-stained T cell-depleted splenocytes from naive C57BL/6 mice, either unloaded, loaded with 1 µM MOG35–55 or MOG CxxCGG40–55. After 30 minutes, cells were gently resuspended, fixed for 10 minutes and analysed by flow-cytometry. Percentage of cells forming conjugates was calculated as described in Figure 1C. Error bars represent 1 SD. (*: *p*<0.05; unpaired t test). Data representative of two experiments. (C) CD4+CD62L+ T cells obtained from 2D2 transgenic mice were polarized by 3 cycles of stimulation under Th17 conditions by addition of TGF-β, IL-6, anti-IL-4, anti-IFN-γ, anti-IL-12 and anti-IL-10 antibodies in the presence of 1 µM MOG35–55. Proliferative response of resting cells (day14) was measured as in (A). Dotted line represents ^3^H-thymidine incorporation of cells incubated with indicated concentrations of MOG 35–55 peptide together with a constant concentration (5 µM) of an irrelevant peptide containing a CxxC motif (CxxC-GG-Dby). (D) The same cells as in (C) were then with DiOC18-stained T cell-depleted splenocytes from naive C57BL/6 mice, either unloaded, loaded with 1 µM MOG35–55 or MOG CxxCGG40–55. After 30 minutes, cells were gently resuspended, fixed for 10 minutes and analysed by flow-cytometry. Percentage of cells forming conjugates was calculated as described in Figure 1C. Error bars represent 1 SD. (*: *p*<0.05; unpaired t test) Data representative of two experiments; (E) Peptide-induced translocation of CD3-ζ and PKC-θ to the immune synapse. Th17 polarized T cells (as in D) were incubated with B cells preloaded with 30 µM MOG35–55 or MOG CxxCGG40–55. After 20 minutes, cells were gently resuspended and added to poly-L-lysine-coated slides for 25 minutes. After fixation and permeabilization, they were stained for CD3-ζ and PKC-θ. Slides were mounted and analysed by confocal microscopy. Between 25 and 75 conjugates from two experiments were analysed. Left panel shows examples of scoring for CD3-ζ and PKC-θ accumulation in conjugates. The results are shown as the average percentage of CD4+ T cells accumulating CD3-ζ and PKC-θ at the contact zone as percentage of total conjugates. White arrows indicate B cells in conjugates. Data representative of three experiments.

To this end, we took the MOG 35–55 peptide encompassing the natural epitope and stimulated the corresponding transgenic TCR cells under conditions of Th1, Th2 or Th17 polarization. Cells were then stimulated with the corresponding peptide containing a CxxC motif. Figure 3A shows that an increased proliferation of Th2 polarized cells was observed when exposed to peptides containing such a motif. Similar results were obtained with a Th1 cell population (data not shown). In Figure 3B, we show that the formation of conjugates between polarized T cells and APCs was enhanced upon exposure to CxxC containing peptides, similarly to what we observed with naïve T cells. Thiol-containing reagents such as β-mercaptoethanol are known to increase cell proliferation [28]. To rule out an interference due to reducing agents in the culture medium, we compared the proliferation of a Th17 polarized cell population following stimulation with a wild type peptide (MOG 35–55) in the presence or not of high concentration of an unrelated thiol-containing peptide (CxxC-GG-Dby). Figure 3C shows that the CxxC motif of the unrelated peptide did not induce proliferation. Figure 3D shows, as for Th2 polarized cells, enhanced conjugate formation between polarized Th17 and APC upon exposure to CxxC peptides.

Figure 3E shows increased translocation and accumulation of PKC-θ and CD3-ζ, characteristics of immune synapse formation, at the interface between the Th17 population and B cells loaded with CxxC peptide.

An explanation to the observed effect on CD4 T cell activation, either naïve or polarized, could still be that insertion of a CxxC motif within the amino terminal flanking region changed the MHC complex binding affinity and half life of the T cell epitope, two parameters known to influence the stimulatory properties of peptide ligands [29]. As mentioned above, the motif lies at distance from MHC class II anchoring residues, and as such, its influence on affinity should be very low, if any. To confirm this prediction, we tested the capacity of MOG35–55 or CxxCGG40–55 to compete with peptide Ea52–68 [30] for binding to class II molecules on APC and, as shown in Figure S3A,B, no difference was observed between the two peptides.

In a displacement experiment wherein the binding of different concentrations of Ea52–68 peptide was measured on splenic B cells preloaded either with MOG35–55 or with CxxCGG40–55, no significant difference was observed between these two conditions and a control consisting in unloaded B cells (Figure S3C).

Taken together, these data suggest that polarized CD4+ T cells, as well as naïve T cells, respond to peptides containing a thioreductase motif by increased proliferation and synapse formation unrelated to changes in MHC binding affinity.

### CxxC-containing class II epitopes increase the level of reduced disulfide bridges of specific CD4 T cell membrane proteins

The redox state of cell surface proteins and the presence of extracellular thiols in the microenvironment are important for T cell activation, and redox-sensitive membrane proteins can present a certain level of reduced disulfifde bridges in the oxidizing extracellular milieu [31], [32].

Activated APC, mainly matured dendritic cells and, to a lower extent, B cells, can secrete thiols, a mechanism mediated intracellularly by thioredoxin [33] (Trx). Trx and other thiol-disulfide oxidoreductases are also active when secreted by DC and can reduce molecules important for T cell functionality, such as surface cytokine receptors [34], integrins [35] or CD4 [36].

As shown with loss of function and wild type peptides in Figures 1, 2 and 3, the potency of CxxC-containing epitopes on cSMAC, proliferation and conjugate formation was dependent on redox activity within the flanking residues. By virtue of its localization outside the MHC cleft, the motif could react with accessible redox-sensitive disulfide bridges at the surface of CD4 T cells during cognate interaction. To determine whether this was the case, resting B cells preloaded with MOG35–55, CxxCGG40–55 or loss of function AxxAGG4055 peptides, were cultured with a MOG35–55 specific Th17 cell line before staining for free T cell surface thiols.

**Figure 4:**
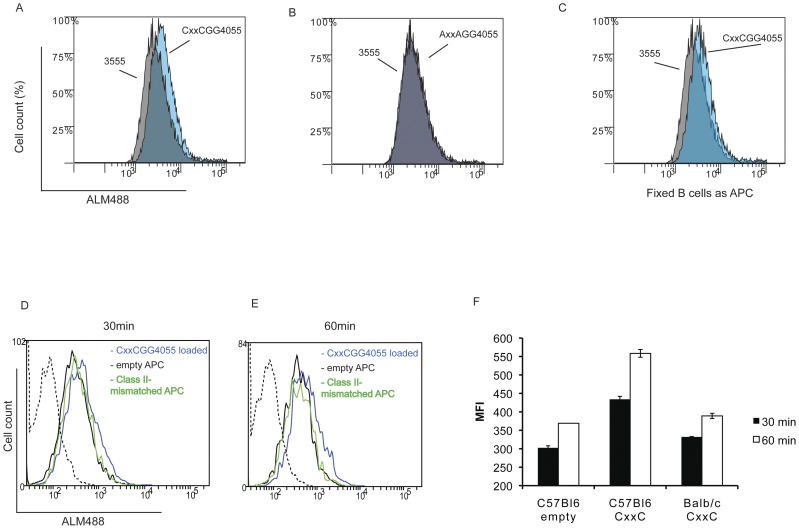
CD4+ T Cell surface thiol level is increased when exposed to CxxC containing peptides. Splenic B cells were purified from C57BL6 mice by depletion, loaded with 50 µM of peptide MOG35–55, CxxCGG40–55 or loss of function AxxAGG40–55, and added to a Th17 cell line specific for MOG 35–55 (ratio APC/T:1/1). After 1 h, cells were washed and stained with Alexa-maleimide 488 (ALM488) and for CD3 and Ia/Ie molecules and analysed by flow cytometry. Blue histograms show ALM488 staining on T cells (CD3 positive/Ia-Ie negative cells) incubated with CxxCGG40–55 (A) or AxxAGG40–55 (B) loaded B cells in overlay with staining on cells incubated with MOG35–55 loaded B cells (grey histograms). (C), same as in (A), but B cells were fixed with glutaraldehyde after loading with CxxCGG40–55. Data representative of three experiments. (D,E) APC (T cell depleted splenocytes) were loaded with 50 µM peptide CxxCGG40–55 (blue line histogram) or kept unloaded (black line histogram) for 2 h at 37°C. After extensive washes, they were added to the same Th17 cells as described before. Histograms show ALM488 staining of CD3 positive/Ia-Ie negative cells after 30 min (D) or 60 min (E) of co-culture. Class II –mismatched APC (BALB/c) loaded with 50 µM peptide CxxCGG40–55 were used as non-specific binding control (green histogram). Dotted histogram is for background fluorescence of CD4+ T cells. Median fluorescence intensity (MFI) obtained for the two time points (D,E) are shown in (F). Representative of two experiments.

Figure 4A shows that the presence of the CxxC motif increased the level of reduced surface thiols when compared with MOG35–55 loaded B cells but not when the loss of function peptide was used (Figure 4B).

Resting B cells were preferred in these experiments because, as previously indicated, activated APC such as DC can release thiols and affect the redox status of surface molecules; besides, we used a short contact time (1 h) between B and T cells, as opposed to more than 6 h required for the detection of APC-released thiols [32]. To further rule out an effect of secreted thiols and released oxidoreductases, we fixed CxxCGG40–55 loaded B cells before co-culture with CD4 T cells. Figure 4C shows that such fixation did not affect cell surface thiol labeling, arguing against any significant effect from secreted factors.

Increased free surface thiols staining was also dependent of cognate recognition of MHC class II –bound CxxCGG40–55 peptide by CD4 T cells, as shown by the lack of increased staining when the CxxC peptide was loaded on class II mismatched APC (Figure 4D, E and F).

To distinguish de novo reduction of disulfide bridges from free surface thiol basal level resulting from CD4 T cell activation, we compared the proliferative response of a Th17 cell line towards either MOG35–55 or CxxCGG4055, after having blocked CD4 T cell surface free thiols with a N-Ethylmaleimide (NEM) [37]. Figure S4 shows that the effect of the CxxC peptide was not altered on NEM-treated CD4 T cells, indicating that CxxCGG4055 was effective by reduction of disulfide bridges, and not by stochastic interaction with free thiols.

Therefore, a CxxC motif located within epitope-flanking residues reduce disulfide bridges at the surface of CD4 T cells, in MHC class II presentation dependent manner.

### Stimulation of naïve or memory CD4 T cells with CxxC containing epitopes influences phenotype even under strong polarizing conditions

The relevance of Th17 cells for many pathologies made it an appropriate case for further examining whether the phenotype of a polarized T cell could be durably altered when exposed to epitopes containing a CxxC motif.

Human Th17 cell induction, upon stimulation with anti-CD3 and anti-CD28, requires low-strength TCR signaling, which can not be reverted even in the presence of pro-Th17 cytokines, such as TGF-β, IL-1β and IL-23 [6]. We therefore wondered whether CxxC motif containing epitopes, by virtue of their strong agonist properties, could not either prevent or reverse the formation of mouse Th17 cells, but, in the present case, in a strict antigen-specific context.

**Figure 5:**
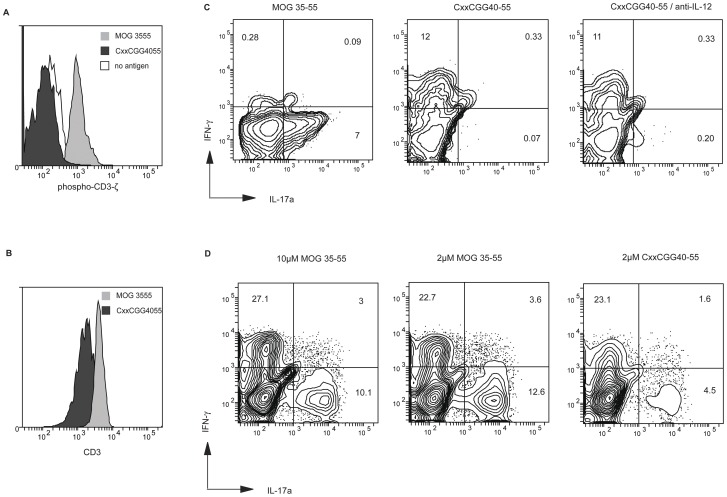
CxxC-containing epitopes prevent and suppress the generation of specific Th17 cells. (A) CD4+CD62L+ T cells obtained from 2D2 transgenic mice were polarized under Th17 conditions with T cell-depleted splenocytes from naive C57BL/6 mice either unloaded (open histogram), loaded with 2 µM MOG35–55 (light grey histogram) or MOG CxxCGG40–55 (dark grey histogram). After 20 minutes, cells were permeated and stained for intracellular phosphorylated CD3-ζ chain. Data are representative of two experiments. (B) The same cells as in (A) were stained for surface CD3 after 18 h of culture with MOG35–55 (light grey histogram) or MOG CxxCGG40–55 (dark grey histogram). (C) On day 6, cells polarized as in (a) and stimulated with MOG35–55 (left panel), modified CxxCGG40–55 (middle) or CxxCGG40–55 with anti-IL-12 antibody (right) were resuspended and restimulated with PMA/ionomycin for 6 h. After permeation, cells were stained for intracellular IL-17 and IFN-γ and analysed by flow cytometry. Numbers indicate percentages of cells in each quadrant. Data are representative of three experiments. (D) A CD4 T cell line was obtained from C57BL6 mice induced into EAE. After three in-vitro stimulations with peptide MOG35–55 in the presence of TGF-β and IL-23, cells were restimulated for three cycles with peptide MOG35–55 either at high concentration (10 µM) or normal concentration (2 µM), or with peptide CxxCGG40–55 (2 µM) in the presence of polarizing cytokines. On day 8 after the last stimulation, cells were tested for intracellular cytokines after stimulation with PMA/ionomycin as in (c).

We stimulated CD4+CD62L+ cells obtained from 2D2 Tg mice in the presence of Th17 polarizing cytokines TGF-β, IL-6 and IL1-β, together with APC loaded with either wild type (wt) MOG35–55 epitope or its CxxCGG40–55 counterpart. Figure 5A shows that, after 60 minutes, cells cultured with MOG35–55 maintained strong phosphorylated CD3-ζ (pY142) level, in contrast to cells stimulated with CxxCGG40–55, which showed even lower pCD3-zeta level than naïve cells incubated in the presence of unloaded APC. Higher TCR degradation was confirmed after 18 h of culture, as shown in Figure 5B when cells stimulated with either peptides were stained for surface CD3. On day 6, cells were stimulated with PMA and ionomycin and stained for intracellular IL-17 and IFN-γ. As shown in Figure 5C (left panel), 7% of cells stimulated with MOG35–55 developed a Th17 phenotype (IL-17(+), IFN-γ(−)), but such phenotype was not detected upon stimulation with CxxCGG40–55 (middle panel).

To prevent interferences by soluble factors during Th17 cell polarization, these experiments were repeated in the presence of antibodies against IFN-γ, IL-4 and IL10, generating the same results (data not shown). We further carried out an experiment in which an anti-IL-12 antibody was added, so as to exclude the possibility of a shift towards an IFN-γ positive/IL17 negative population due to APC-derived IL12 [38] (Figure 5C; right panel).

Stimulation with CxxC-containing epitopes also deeply influenced naïve CD4+ cell maturation under TH17 conditions, as illustrated by the lack of CD62L expression already observed after a single stimulation, in contrast to stimulation with nominal epitope 35–55 (Figure S5).

These data indicate that stimulating naïve 2D2 MOG-specific CD4+ T cells with a strong specific agonist blocked the generation of Th17 cells even under stringent induction conditions. We then turned to MOG35–55 specific polyclonal memory cells obtained from mice induced into EAE, which contain IL-17 positive, IFN-γ positive and double positive cell populations [39]. Figure 5D shows that such in vivo polarized cell populations could be modified by three cycles of stimulation (illustrated here with cells in the presence of TGF-β and IL-23) with a CxxC-containing MOG peptide, with a significant reduction in the number of IL-17 producing cells (Figure 5D, right panel), an effect which could not be obtained when increasing the concentration of MOG35–55 epitope (Figure 5D, left panel).

### Peptides containing a CxxC motif elicit the transformation of naïve or polarized CD4+ T cells into potent cytolytic cells inducing apoptosis of cognate antigen-presenting cells

We next evaluated whether the increase in proliferation observed with both naïve and polarized cells altered the functional properties of such cells, with acquisition of cytolytic properties. We measured induction of apoptosis of B cells or DC used as APCs, by evaluating either caspase-3 activation, annexin V binding or by using the Tunel assay. The initial G121 CD4+ T cell clone induced such apoptosis by Fas-FasL interactions [8].

Examples of such apoptosis induction are shown in Figure S6A,B, using a CD4+ T cell clones (R3TB7) with cytolytic properties and obtained from independent immunizations with p21–35. Figure S6A shows experiments carried out with p21–35-loaded CD11c+ dendritic cells (left panel) or the B cell line WEHI-231 (right panel). Upon incubation with R3TB7 virtually all DC or WEHI-231 cells entered into apoptosis. Again, the same experiments carried out with Annexin V showed identical results (not shown).

Apoptosis can be induced by either the Fas-FasL pathway or by secretion of cytotoxic granules containing granzymes [40]. We therefore attempted to inhibit each of these pathways. Figure S6B (left panel) indicates that addition of increasing concentrations of an antibody towards FasL, or of granzyme B inhibitors (right panel), to a cell culture containing p21–35 loaded WEHI-231 cells and R3TB7 increased the number of surviving WEHI-231 cells in a dose-dependent manner. In a number of additional experiments we showed that the anti-FasL antibody restored up to 80% of WEHI-231 cell survival, whereas no significant restoration of survival was obtained with granzyme B (GZB) inhibitor z-AAD-fmk (Figure S6B, right panel), indicating that GZB did not account for much of the cytolysis of B cells. In further experiments, we used EGTA as an inhibitor of granule exocytosis, which also showed essentially no restoration of WEHI-231 cell survival (data not shown). Metalloproteases are known to cleave surface FasL and its release in cell environment possibly mediates apoptosis [41]. When cytolytic assays were repeated in the presence of different concentrations of a metalloprotease inhibitor (GM6001), apoptosis was not decreased, suggesting that killing was not due to a paracrine effect (Figure S6B, left panel).

We concluded that apoptosis required cognate recognition of pMHC complexes at the surface of the APC by cells pre-exposed to peptides containing a CxxC motif. To further establish that apoptosis required direct synapse formation, we carried out an assay in which peptide-loaded CD11+ dendritic cells were added to the two compartments of a transwell culture system and a CD4+ T cell clone (G121) added to only one compartment. As expected, apoptosis was observed in 89% of the cells in direct contact with the CD4+ T cell clone, as measured by Annexin V expression (Figure S6C, left panel), whilst only 15% of dendritic cells died in the culture compartment in which no T cells were added (Figure S6C, right panel), a value comparable to that obtained by culturing dendritic cells alone (not shown).

**Figure 6:**
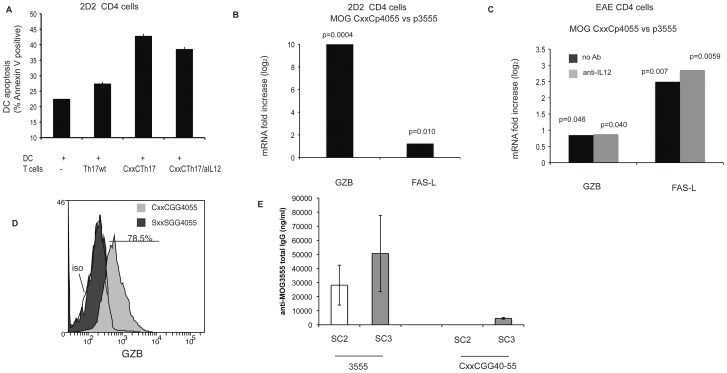
Induction of cytolytic factors in CD4 T cells stimulated with CxxC modified peptides. (A) CD4+CD62L+ cells from 2D2 transgenic mice stimulated for three cycles under Th17 polarizing conditions with wt or modified peptide (as in Figure 5) were added to LPS activated dendritic GFP-transduced JAWS II cells (ratio T/DC: 2/1) loaded with peptide MOG 35–55. After 20 h, apoptosis of JAWS cells was measured by Annexin V staining of GFP positive cells. Error bars respresent 1 SD. (***p = 0.0018; NS *p* = 0.02). Two tailed *p* values are from unpaired t test. (B) quantitative PCR for FAS-L and GZB on polyclonal 2D2 CD4 cells differentiated under TH17 conditions with wild type or CxxC peptide (same cells as in Figure 5A). Fold change for CxxC-GG40–55 cells relative to MOG35–55 generated cells was determined after stimulation with APC loaded wild type MOG35–55. Results were normalized to 18S rRNA. Two tailed *p* values are derived from unpaired t tests. (C) Quantitative PCR on EAE CD4 cells after 3 cycles of in vitro amplification (in the presence of IL-23 and TGF–β) with MOG35–55 or CxxCGG40–55. Fold change for CxxCGG40–55 cells relative to MOG35–55 generated cells was determined after stimulation with T cell depleted splenocytes loaded wild type 35–55. Grey histograms are for CxxCGG40–55 stimulated in the presence of an anti-IL-12 antibody. Results were normalized to 18S rRNA. Results are representative of two independent experiments. Two tailed *p* values are derived from unpaired t tests. (D) CD4+CD62L+ cells from 2D2 transgenic mice were expanded for three cycles with CxxCGG40–55 or loss of function SxxSGG40–55. Ten days after the third stimulation, cells were stimulated with MOG 35–55 for 18 h and stained for intracellular GZB. Grey histogram is for cells expanded with CxxCGG40–55 and black histogram for SxxSGG40–55 expanded cells. Open histogram is for isotype control staining (iso). Data representative of two experiments. (E) C57BL/6 mice were immunized by 3 footpad injections of 10 µM MOG3555 or CxxCGG40–55 peptides in Incomplete Freund’s adjuvant at 11 days intervals. Ten days after the second (SC2) and third (SC3) injections, mice (n = 5 in each group) were bled and the presence of anti-MOG35–55 IgG antibodies was detected in a quantitative Elisa assay.

We extended these investigations to transgenic TCR CD4+ T cells. Figure 6A shows that after 10 days, 2D2 Tg naïve cells stimulated with a specific CxxC-containing epitope acquired killing properties for dendritic cells loaded with MOG35–55 epitope, a finding not observed with 2D2 Tg cells stimulated with the nominal epitope. The figure also shows that this effect was observed even in the presence of an anti-IL-12 antibody under Th17 polarizing conditions (same cells as in Figure 5C).

GZB and FAS-L gene expression was assessed by qPCR on the cells described above after stimulation with APC loaded with p35–55. Both genes were transcribed at significantly higher rate in cells exposed to CxxCGG40–55, as compared to MOG35–55 epitope, with GZB showing the strongest increase (Figure 6B). This was confirmed at protein level by intracellular staining for GZB (Figure 6D). Figure 6C shows that increase in lytic factors was also observed in memory cells derived from EAE mice (described in Figure 5D), but, interestingly, the highest increase was observed for Fas-L.

In vitro data obtained with peptide antigens do not necessarily correlate with in vivo immunogenicity [29]. The in vivo effect of CxxC-containing epitopes was tested by immunizing mice three times with MOG35–55 or with CxxCGG40–55 peptides in incomplete Freund’s adjuvant. Ten days after the second and the third injection, serum was tested for total IgGs against MOG peptides. Figure 6E shows that specific IgGs were barely detectable even after three subcutaneous immunizations, contrasting to the high IgG concentrations obtained with wild type peptide MOG35–55 already after two injections. This suggests that immunization with CxxCGG40–55 peptide induced a population of CD4 T cells able to kill B lymphocytes in vivo, suppressing the production of antigen-specific antibodies.

Thus, the increase in proliferation observed after exposure to thioreductase-modified peptides correlated with acquisition of apoptosis induction properties on APC, independently of the fact that cells were or were not polarized.

Altogether, we concluded that naïve as well as polarized CD4+ T cells, which had been exposed to cognate peptides containing a CxxC motif, acquired the capacity to induce apoptosis of DC or B cells by a mechanism requiring the formation of an immune synapse with pMHC class II complexes, even when the peptide encompassed only the natural epitope sequence. Fas-FasL interaction and GZB significantly participated to these newly acquired properties.

## Discussion

We show in the present paper that activation of naïve as well polarized CD4+ T cells with peptides encompassing a thiol-disulfide oxidoreductase motif within flanking residues of natural MHC class II-restricted epitopes alters the functional properties of such CD4+ T cells with acquisition of potent cytolytic activity. Cytolytic CD4+ (cCD4+) T cells then induce apoptosis of APC after cognate recognition of corresponding natural sequence peptides. These properties result from prolonged IS formation with increment in proliferation.

Monocysteinic glutaredoxin motif (CxxS) can exert a nucleophilic attack on target protein disulfide bridges and thereby create stable intermediates [34]. The synapse formed between APC and CD4+ T cells contains several proteins carrying disulfide bridges, which could be utilized to change the kinetic properties of the synapse and subsequent signaling in CD4+ T cells. The observation that changing the strength of synapse formation could profoundly alter the properties of CD4+ T cells [42], recently illustrated by the requirement of low-strength synapse formation for Th17 polarization [6], prompted us to reconsider observations made on a T cell clone derived from a common allergen [8].

Converting the natural CxxS motif located in flanking residues of the class II-restricted p21–35 recognized by such clone into a full thiol-disulfide oxidoreductase motif (CxxC) could reinforce the properties observed with the initial clone.

Experiment results reported in the present paper demonstrate that this prediction was correct. The number and the duration of dimers formation between APC and CD4+ T cells increased significantly by altering the CxxS motif into a CxxC format, with striking increase in CD3 catabolism, a hallmark of synapse strength [22].

Naïve and polarized CD4+ T cells can be converted into cytolytic T cells by exposure to CxxC motif-containing epitopes, at least as we can conclude from the two different model systems used here, namely class II-restricted epitopes from an airborne allergen, and the myelin oligodendrocytic glycoprotein.

Our data not only confirm observations showing that the quality of the signal provided to CD4+ T cells at the IS level, in the absence of cytokines, determines cell phenotype [42], but extend these to polarized CD4+ T cell subsets. Indeed, bona fide Th1, Th2 as well as Th17 cells can be converted into cytolytic cells, loosing initial properties to adopt a seemingly uniform phenotype dominated by acquisition of cytolytic properties. Activation by epitopes containing a CxxC motif seem therefore sufficient enough to override the transcriptome and epigenetic signatures associated with polarized effector cells. In keeping with this, Lee et al [38] have shown that even polarized cells keep bivalent genetic markers, which may allow cells to switch to an alternative phenotype when appropriate conditions are provided. The concept of CD4+ T cell plasticity covers polarized CD4+ T cells in addition to naïve cells.

Th17 cells have attracted much attention due to their involvement in many pathologies [43]. We therefore examined in further details whether acquisition of cytolytic properties was paralleled with loss of pathogenic properties such as production of IL-17. Under polarizing conditions, even when such conditions were maintained during activation with CxxC motif containing epitopes, we observed a gradual loss of IL-17 production. These data were obtained with both TCR transgenic cells and with polyclonal CD4+ cell populations, establishing their physiological relevance. They also extend findings reported recently on human cells and loss of a Th17 phenotype by increasing the strength of TCR stimulation [6]. In fact, our data establish as a general rule that increased synapse strength induces a cytolytic phenotype, whatever the initial status, naïve or polarized CD4+ T cells. It remains to be determined whether alternative subsets of effector T cells, such as Th9 cells and Tfh can also be directly converted into cytolytic T cells.

APC were induced into apoptosis by a mechanism requiring cell-cell contact. An obvious candidate for such an activity is the Fas-FasL pathway, and we observed up to 80% inhibition of apoptosis using an anti-FasL antibody. Yet, cCD4+ T cells express surface FasL and the release of FasL requires the presence of a metalloprotease [41]. In the light of conflicting observations on the passage of soluble FasL trough a transwell membrane, we used an inhibitor of metalloprotease to discard a significant involvement of soluble FasL into the apoptosis process.

Another mechanism by which apoptosis can be induced is by exocytosis through the synapse [44] of cytotoxic granules, which contain soluble FasL and serine proteases including granzymes [45]. We observed that both perforin (data not shown) and granzyme B were increased at transcription level in cCD4+ T cells. Perforin and GZB synergize facilitating the penetration of GZB. However, addition of EGTA, an inhibitor of perforin, did not reduce apoptosis. Granzymes can penetrate target cells in the absence of perforin though the precise mechanism is not fully understood [46]. However, addition of Z-AAD-fmk, an inhibitor of GZB did not prevent induction of apoptosis. This inhibitor is toxic for cells when used at higher concentrations and the demonstration that GZB does not participate in apoptosis induction therefore awaits further investigations. Involvement of the Fas-FasL pathway does not preclude the participation of GZB, the two mechanisms being convergent, as FasL activates caspase 8, itself an activator of caspase 3, which is directly activated by GZB [46].

Noteworthily, induction of APC apoptosis was observed with dendritic as well as with B cells, suggesting that both primary and secondary immune responses should be amenable to suppression.

Conditions under which cCD4+ T cells are elicited in vivo, in our expertise, seem to vary according to the adjuvant used. Thus, immunization in CFA/IFA provided cytolytic Th1 like cells, that were difficult to expand due to their higher sensitivity to activation-induced cell death [47], whereas cells obtained by immunization in alum with CxxC-containing peptides expressed full cytolytic activity directly ex vivo and could be easily expanded in vitro. Cytolytic activity in CD4+ T cells was usually associated with the Th1 phenotype, but a recent report suggested that cytolytic activity could also be detected in Th0 and, to a lower extent, in Th2 cells [48]. In the same report, antigen dose was demonstrated to modulate cytolytic activity, confirming our present data on enhanced TCR triggering leading to the acquisition of lytic capacity by CD4+ T cells. We are now working on the phenotypic characterization of cytolytic CD4+ cells (Carlier *et al* in preparation), but as shown in Figure S5, the phenotype of naïve cells was strongly influenced in our system, opposed to data obtained in the above report where no correlation was observed between cytolytic capacity and differentiation [48].

Soluble factors can induce lytic properties in CD4+ T cells, under somewhat artificial culture conditions, such as stimulation in the presence of high IL-2 concentration [48] or incubation with both IL-10 [49] and IFN-α. Dual costimulation with agonist antibodies to CD134 and CD137 has recently been shown to convert CD4+ T cells into cytolytic cells, with induction of Eomes, a transcription factor associated with CD8+ lytic function, and expression of GzmB [50]. To note, however, none of these conditions elicited only antigen-specific cytolytic CD4+ T cells.

One important characteristics of the present approach is that, in contrast to altered peptide ligands (APLs), class II epitopes are modified outside and at distance (a linker of up to 4 aminoacids) from the MHC class II binding cleft. This provides the possibility of activating naïve CD4+ T cells directly relevant to pathogenesis and, most importantly, to alter the phenotype of pre-existing effector CD4+ T cells, at least Th1, Th2 cells and Th17 cells. This should also prevent the somewhat unpredictable effects observed with APLs, which put a number of trials to an end [51]. Worth reminding, however, is the fact that in the present study we actively induced a population of peptide-specific CD4+ T cells using an adjuvant, while APLs are administered either by the mucosal or systemic route in the absence of adjuvant [52].

In addition, elimination of APC by induction of apoptosis prevents activation of alternative CD4+ T cells specific for additional T cell epitopes of the same antigen, providing a means by which responses towards complex antigens can be prevented or suppressed.

In principle, any class II restricted T cell can be switched to a cytolytic phenotype upon cognate recognition with an epitope containing a CxxC motif. MHC class II determinants are open both sides and can accommodate peptides of up to 20 aminoacids, including the sequence of 9 aminoacids inserted into the class II cleft [53]. The present data therefore provides the background for a strategy by which it may be possible to prevent or suppress unwanted immune responses. This is obviously contingent to the understanding of cCD4+ properties in vivo, including tissue distribution.

## Experimental Procedures

### Ethics Statement

Mice were treated in accordance to european regulations and experiments were approved by University of Leuven Ethical Committee (Project LA1210196/P1432008).

### Peptides

Peptides were synthesized by solid phase FMOC chemistry (Eurogentec) (purity >85%). Sequences of wild type and modified peptides from *Dermatophagoides pteronyssinus* group 2 allergen epitope p21–35, ovalbumin epitope 323–339, MOG epitope 35–55 and alloantigen Dby epitope 605–619 are indicated in Table 1 with their respective MHC Class II anchoring residues.

**Table 1:** Peptide sequences.

				P(−4)	P(−3)	P(−2)	P(−1)	P1	P2	P3	P4	P5	P6	P7	P8	P9	P10						
**P2135**	**wt**			C	H	G	S	E	P	C	I	I	H	R	G	K	P	F					
	**CxxCp2135**			C	H	G	C	E	P	C	I	I	H	R	G	K	P	F					
	**P(−4)A**			A	H	G	S	E	P	C	I	I	H	R	G	K	P	F					
	**P(−1)A**			C	H	G	A	E	P	C	I	I	H	R	G	K	P	F					
	**AxxAGGp2135**	A	H	G	A	G	G	E	P	C	I	I	H	R	G	K	P	F					
	**CxxCGGp2135**	C	H	G	C	G	G	E	P	C	I	I	H	R	G	K	P	F					
**MOG**	**wt 35–55**		M	E	V	G	W	Y	R	S	P	F	S	R	V	V	H	L	Y	R	N	G	K
	**CxxCGG40–55**	C	G	P	C	G	G	Y	R	S	P	F	S	R	V	V	H	L	Y	R	N	G	K
	**AxxAGG40–55**	A	G	P	A	G	G	Y	R	S	P	F	S	R	V	V	H	L	Y	R	N	G	K
	**SxxSGG40–55**	S	G	P	S	G	G	Y	R	S	P	F	S	R	V	V	H	L	Y	R	N	G	K
**OVA**	**wt 323–339**	I	S	Q	A	V	H	A	A	H	A	E	I	N	E	A	G	R					
	**CxxCGG329–339**	C	G	H	C	G	G	A	A	H	A	E	I	N	E	A	G	R					
	**AxxAGG329–339**	A	G	H	A	G	G	A	A	H	A	E	I	N	E	A	G	R					
**Dby**	**wt 605–619**	G	S	A	N	A	G	F	N	S	N	R	A	N	S	S							
	**CxxCGG 611–619**	C	H	G	C	G	G	F	N	S	N	R	A	N	S	S							

### Fluorescence-based assay for redox activity

A FITC –NH-Gly-Cys-Asp-COOH peptide was synthesized (Eurogentec) and self-quenched by solubilization in DMSO ((FITC-Gly-Cys-Asp)_ox_). The reduction of 2.5 µM (FITC-Gly-Cys-Asp)_ox_ was followed on a 96 well plate during 40 minutes (25°C) after incubation in PBS with peptides (25 µM) or 2 mM Dithiothreitol (DTT) as previously described [20]. Reduction was measured by increase in fluorescence at 530 nm after excitation at 494 nm on a CytoFluor® multiplate reader (Applied Biosystems).

### Mice

BALB/c mice were from Taconic. 2D2 TCR Tg (Tcra2D2,Tcrb2D2)1Kuch/J on a C57BL/6 background and BALB/c OVA TCR D011.10 mice (C.Cg-Tg(DO11.10)10Dlo/J) were purchased from The Jackson Laboratory. Marilyn female mice, expressing a TCR specific for Dby-encoded HY peptide (residues 605–619) on a C57BL/6 RAG2−/− background were a generous gift from Prof. Michel Braun (IMI ULB, University of Brussels).

### Cell culture

Dendritic cells (DC), T cells and B cells were cultured in RPMI 1640 medium containing 5% FCS, 50 µM 2-ME, 200 µg/ml Gentamicin (Invitrogen). WEHI 231 cells and JAWS II cells (C57BL/6 dendritic cell line) were purchased from the European collection of cell cultures (ECACC).

### Preparation of T cells and antigen presenting cells - APC

Naïve CD4+ cells from 2D2 transgenic, DO11.10 or Marilyn mice were purified by immunomagnetic negative selection (CD4+CD62L+ T cell isolation kit II; Miltenyi) following manufacturer instructions. For the preparation of APC, T cells from splenocytes of BALB/c or C57BL/6 mice were removed by immunomagnetic depletion with CD90 microbeads (Miltenyi). CD11c DC were obtained by immunomagnetic selection (CD11c microbeads; Miltenyi). JAWS II cells were transduced for expression of GFP as previously described [54].

### Derivatization of cytolytic CD4+ T cell clones

BALB/c mice were immunized by 3 footpad injections of 20 µg/ml peptide p21–35 in alum at 2 weeks intervals. Ten days after the last injection, spleen CD4+ T cells (CD4 T cell isolation kit, Miltenyi) were stimulated with Mitomycin-C (Kyowa) treated T cell depleted splenocytes from naïve mice in the presence of peptide p21–35 (2 µM). After 10 days, cells were restimulated under the same conditions but with 10 U/ml mouse IL-2 (Roche). After the fifth restimulation, T cells were subcloned in the presence of 10 U/ml IL-2 by limiting dilution. Subsequent specific stimulations were carried out in the presence of 10 U/ml mouse IL-2. The G121 T cell line was derived as previously described [8].

### Derivatization of polarized T helper cells

Polarized Th1 and Th2 populations from TCR transgenic mice were obtained by stimulating naïve CD4+CD62L+ cells with T cell depleted splenocytes loaded with corresponding peptides and in the presence of 20 µg/ml anti-IL-4 (11B11), 20 ng/ml IL-12 (Biosource) or with 5 ng/ml IL-4, 20 µg/ml anti-IFN-γ (XMG1.2) and 20 µg/ml anti-IL-12 (C17.8), respectively. Cells were stimulated for three cycles under these conditions in the presence of 10 U/ml IL-2. Th17 cells were prepared from 2D2 splenic naïve CD4 cells stimulated with APC loaded with 2 µM MOG 35–55 peptide in the presence of 50 ng/ml IL-6, 5 ng/ml TGF-β, 10 ng/ml IL-1β, 20 µg/ml anti-IL-4 and 20 µg/ml anti-IFN-γ. They were maintained with 5 ng/ml TGF-β for successive restimulations. All cytokines were from Biosource except TGF-β (R&D).

### Conjugate analysis

APC were stained with 1 µM DiOC_18_ membrane stain (Invitrogen) and loaded for 1 hour with 1 µM of indicated peptides. They were then added to CD4+ cells (ratio 1∶1) stained with 1 µM CellTrace™DDAO SE (Invitrogen) followed by a short centrifugation to favor cell contact (stainings were done in PBS-0.1%BSA for 15 minutes at 37°C). At indicated times, cells were gently resuspended and fixed for 10 minutes at 25°C with 4% paraformaldehyde and analysed on a FACSCantoII flow cytometer (BD Biosciences). Conjugates were measured as the ratio of DDAO SE/DiOC_18_ dual positive population to total DDAO SE positive cells.

### Proliferation of CD4+ cells

CD4+ cells from different mouse strains were cultured for 4 days with mitomycin C treated CD90 depleted splenocytes (APC) from the respective haplotype with indicated amounts of peptides. (^3^H)-thymidine (0.5 µCi) was added for the last 18 hours before scintillation counting. Where indicated, CD4 T cells were incubated for 35 minutes on ice with 5 µM N-ethylmaleimide (NEM; Sigma). Reaction was quenched during 5 minutes on ice with 10 µM Glutathione (Sigma). After washing, CD4 cells were added to APC as indicated above.

### TCR Downregulation

MOG 35–55 specific CD4+ T cells were incubated for indicated timing with APC (ratio 1∶1) preloaded with 1 µM wt peptide 35–55 or CxxC peptide. Cells were then resuspended vigorously to break conjugates and stained with FITC-labeled anti-CD4 and PE-labeled anti-CD3 antibodies, clones RM4.5 and 17A2, respectively (BDPharmingen). CD4 positive cells were then analysed by flow cytometry (FACSCalibur, BD Biosciences) for surface expression of CD3.

For p21–35 specific clone, cells were pretreated for 1 h with 10 µg/ml cycloheximide (Sigma) and incubated for 1 h with WEHI-231 B cells preloaded with 50 µM of indicated peptides. Cells were resuspended, washed with PBS-EDTA 2 mM, and stained with APC-H7-labeled anti-CD4 (RM4–5;BDBiosciences), APC-labeled anti-CD3-ε (145-2C11; eBioscience) or PE-labeled anti-TCR-β (H57–597; BDBiosciences) antibodies.

### Surface thiols detection

CD4 T cells were cultured for indicated times with B cells purified by negative selection (B cell Isolation Kit, Miltenyi) or with T cell depleted splenocytes where indicated. Cells were collected, washed with PBS-EDTA and vigorously pipetted to break cell agregates. They were then stained with 5 µM Alexa-488 maleimide (ALM488; Invitrogen) for 15 minutes on ice. After washing with cold PBS to remove unincorporated ALM488, CD4+ T cells were stained for CD3 (17A2, BDPharmingen) and I-Ab/I-Eb (M5/114.15.2, BDPharmingen). After 20 minutes on ice, cells were washed with cold PBS, fixed with paraformaldehyde (4%) and analysed on a FACSCantoII flow cytometer (BD Biosciences). Signal for ALM488 was analysed on CD3 positive/I-Ab-IEb negative cells. Where indicated, B cells were fixed with 1% glutaraldhyde (Sigma) and washed before coculture with CD4+ T cells.

### Assessment of Ea52–68/I-Ab complexes

Splenic B cells were purified (B cell Isolation Kit; Miltenyi), treated for 30 min with 20 mM sodium azide and 50 mM 2-deoxy-D-glucose to block recycling of MHC class II molecules and used for MHC class II binding with peptides. For competition assay, Ea52–68, a self peptide derived from H2E molecule [55], was used at 25 µM together with variable concentrations of MOG35–55 or CxxCGG40–55 peptides. After 1 h, cells were washed and incubated with a FITC-stained monoclonal antibody (Y-Ae) recognizing Ea52–68/I-Ab complex prior to fixation with paraformaldhyde (4%) and analysis on a FACSCantoII flow cytometer (BD Biosciences). For displacement assay, B cells were first loaded at 37°C with 10 µM of MOG peptides as indicated. After washing, a titration curve of Ea52–68 peptide was added for 1 hour before washing and detection with Y-Ae Ab as indicated above. In this assay, a preloaded peptide ligand will be displaced by adding Ea52–68 peptide as a function of its affinity or half-life. Unloaded B cells are used to determine reference signal for Ea52–68 binding.

### Apoptosis detection

Annexin V-FITC or -PE and 7-AAD were used to detect cell death in B cells, dendritic cells (DC) and T cells (Annexin V detection kit, BD Biosciences). In some experiments, apoptosis was measured by intracellular detection of activated caspase-3 with FITC- or PE-labelled antibodies (Pharmingen) according to manufacturer's instructions. GFP-transduced JAWS II DC were activated for 24 h with 5 µg/ml LPS. After 4 extensive washes, they were added to CD4 cells and co-cultured for 20 h in the presence of peptide antigen before staining with Annexin V-APC (Immunotools) and analysed by flow cytometry.

For inhibition of GZB activity, Z-AAD-fmk (Calbiochem) was added at indicated concentrations during the entire co-culture period. Inhibition of soluble FasL release was performed during the co-culture period with the metalloprotease inhitor GM6001 (Sigma) at indicated concentrations.

### Detection of CD3ζ phophorylated proteins

CD4+ T cells from 2D2 tg mice (10^6^ cells) were purified as previously described and stimulated with T cell depleted splenocytes from C57BL/6 mice loaded with 50 µM of indicated peptides (ratio 1∶3) for 60 minutes. Cells were then immediately fixed with 20 volumes of pre-warmed BD™Phosflow Lyse/Fix buffer (BDbiosciences) for 10 minutes at 37°C. After washing with PBS, cells were permeated overnight with 200 µl of BD™Phosflow Perm Buffer III (BDbiosciences) at −20°C. Cells were then washed twice with PBS containing 1% FBS and blocked for 20 minutes at room temperature with BD FcBlock™ (0.5 µg/10^6^ cells). Antibodies against CD4-FITC (clone RM4.5) and CD3ζ-APC (pY142) (clone K25-407.69) (BDbiosciences) were then added according to the manufacturer instructions for 30 minutes at room temperature. After two final washes, cells were analysed on a FACSCantoII flow cytometer (BD Biosciences).

### Detection of immune synapse formation

CD4+ T cells were mixed with peptide loaded B cell blasts (pre-activated with 5 µg/ml LPS for 24 h) at a ratio 1∶2, centrifuged at 200 g for 5 minutes and incubated for 20 minutes at 37°C. Cells were then transfered to poly-L-Lysine coated slides for 25 minutes at 37°C. Cells were then fixed with 4% paraformaldehyde and permeabilized with wash buffer (saponin 0.1%, Hepes 20 mM, PBS pH7.2) for 30 minutes. Slides were blocked for 18 h with 5% goat serum in wash buffer (Dako) and incubated for 2 h at 4°C with antibodies anti-CD3-ζ (6B10.2; Santa Cruz) and anti-PKC-θ (C-18; Santa Cruz) in wash buffer containing 2% BSA. This was followed by goat anti-mouse Alexa-546 (for CD3) or goat anti-rabit Alexa-568 (Invitrogen) for 1 h at 25°C. Slides were then analysed on a Zeiss CLM510 confocal microscope.

### Detection of MOG 35–55-specificic antibodies

C57BL/6 mice were immunized with 10 µM of MOG35–55 peptide or CxxCGG40–55 peptide emulsified in incomplete Freund’s adjuvant (50 µl per footpad) for three consecutive injections (11 days interval). After the second and third immunization, mice were bled and serum prepared by standard centrifugation. 1∶100 and 1∶200 dilutions of each serum were done and 100 µl of samples were added in duplicate to an Elisa plate (Nunc™) precoated with a mixture of MOG35–55 and CxxCGG40–55 peptides (1 µg/ml each; PBS). A monoclonal IgG antiboby specific for MOG35–55 was used as standard (Alpha Diagnostic). After 2 h, plates were washed and an horseradish peroxidase-conjugated goat anti-mouse IgG antibody was added for 1.5 h (0.5 µg/ml; Biorad). Plates were washed and antibodies detected with TMB substrate (BD Biosciences) after reading at 450 nm on a Microplate Reader (Molecular Device).

### Detection of Granzyme B

Intracellular staining for GZB was done with Cytofix-Cytoperm and Perm-Wash buffers (BD Biosciences) following manufacturer’s instructions (clone 16G6). Samples were acquired on a FACSCantoII flow cytometer (BD Biosciences).

### Gene expression analysis

mRNA was extracted from cells with RNeasy® mini kit (Qiagen) and cDNA was synthesised with RT2 First Strand Kit® (SABiosciences). PCR reactions were done with RT2 qPCR Primer Assay® with primer pairs for Gzb and Fas-L (all from SABiosciences) and run on an ABI 7500 instrument. Samples were run in triplicates. 18S rRNA was used as normalization factor.

### Statistical analysis

Non-parametric tests (Mann-Whitney and one-way anova) were used for evaluating significance with Prism software. For significance of QPCR data, unpaired t tests were done with SABiosciences Data Analysis template V3.3 (Qiagen).

## Supporting Information

Figure S1 **Effects of aminoacid residue substitution in the p21–35 flanking sequence.** A p21–35 specific cytolytic CD4+ clone (G121) was cultured with T cell depleted mitomycin C-treated splenocytes in the presence of 0.1 µM of either natural sequence peptide p21–35 (CxxS motif), CxxCp2135 peptide (CxxC motif) or CxxCGGp21–35 (CxxCGG motif, with a linker made of two glycine residues). ^3^H-thymidine incorporation was measured after 72 h. Data representative of two experiments. Similar results were obtained with an alternative p21–35 specific cCD4+ clone R3TB7. Error bars represent 1 SD; One way Anova analysis of variance was done for comparing the three peptides (NS, *p* = 0.12).
(TIF)

Figure S2 **T cell epitopes containing a thioredoxin active motif reduce disulfide bridges in vitro.** The redox capacity of peptides CxxSp21–35 or CxxCp21–35 (25 µM) was tested by time course experiment after incubation with self-quenched redox substrate (FITC-Gly-Cys-Asp)_ox_. The reaction was followed for 40 minutes after excitation at 495 nm and recording FITC emission at 530 mm. Dithiothreitol (DTT; 2 mM) was used as positive control.
(TIF)

Figure S3 **Insertion of a CxxC motif within flanking residues does not modify peptide affinity for MHC class II molecules.** (A) Competition experiments for MHC class II binding were carried out by incubating B cells for 1 h at 37°C with Ea52–68 peptide (25 µM in each experiment), either alone (black histogram) or together with peptides MOG 35–55 (red histogram) or CxxCGG40–55 (blue histogram) at 10 µM (left panel) or 0.5 µM (right panel). After washing, binding of peptide Ea52–68 was detected with a fluorescent antibody (Y-Ae) recognizing the peptide Ea52–68/MHC class II complex. Left and right panel represent detection of the complex in the presence of 10 µM or 0.5 µM of competing peptides, respectively. (B) Decrease in signal was calculated from data obtained in (A). Non-parametric test (Mann-Whitney) showed no difference between peptides (p>0.3). Error bars show mean +/− SD. (C) B cells (5.10^4^ cells/test) were loaded during 1 h with 10 µM of peptide MOG 35–55, 10 µM of peptide CxxCGG40–55, or kept unloaded (neg). After washing, cells were incubated with Ea52–68 peptide at different concentrations. After 1 h, cells were washed and peptide displacement was assessed by detecting Ea52–68/class II complex as in (A) (NS; *p* = 0.2) One-way anova test was used to compare the three curves. Error bars show mean +/− SD.
(TIF)

Figure S4 **The effects resulting from inserting a CxxC motif do not depend on interaction with free surface thiols.** A Th17 CD4 T cell line obtained from mice induced into EAE was stimulated with APC in the presence of MOG35–55 or CxxCGG40–55 peptides for 48 h. Where indicated, CD4 cells were pre-treated with 5 µM N-ethylmaleimide (NEM) before culture. ^3^H-thymidine was added for the last 12 h of culture. Error bars represent 1 SD. Data representative of two experiments.
(TIF)

Figure S5 **Increased maturation of naïve CD4+ T cells exposed to a CxxC-containing peptide.** Naïve cells from 2D2 transgenic mice were polarized under Th17 conditions and analyzed for CD62L expression after a single stimulation (day 10) with 1 µM MOG CxxCGGp40–55 (grey histogram) or with 1 µM MOG 35–55 (black histogram). Open histogram is for isotype control antibody. Data representative of two experiments.
(TIF)

Figure S6 **Induction of apoptosis in APC requires direct cell recognition and is mediated by Fas-L.** (A) CD11c+ dendritic cells (DC) were obtained from BALB/c splenocytes by positive selection using magnetic beads and activated by LPS. DC (left panel) and WEHI-231 cells (right panel) were then loaded with 1 µM p21–35 peptide and co-cultured for 18 h with R3TB7 cCD4+ T cell (black areas). Open histograms represent anti-cleaved caspase-3 staining in DC or B cells cultured without T cells. Detection of cleaved caspase 3 expression was taken as a marker of cell apoptosis. Data is representative of a minimum of 3 independent experiments. (B) DiOC18 stained WEHI-231 B cells were loaded with 1 µM p21–35 peptide and incubated with R3TB7 (1/1 ratio) in the presence of increasing concentrations of anti-FasL antibody or of the metalloprotease inhibitor GM6001, as indicated (left panel). Increasing concentrations of an antagonistic peptide of GZ-B (Z-AAD-fmk) were also tested (right panel). Apoptosis of WEHI-231 cells was measured after 18 hours and staining with Annexin V and 7AAD. Error bars represent 1 SD. Data representative of two experiments. (C) CD11c+ DC were loaded with p21–35 peptide and incubated in the presence of cCD4+ G121 T cell clone for 18 h at a 1/1 ratio. The left panel shows DC apoptosis measured by Annexin V binding (gated on Vβ8 negative cells). The right panel shows the same experiment but with peptide-loaded DC and cCD4+ G121 cells separated by a semi-permeable membrane in a Transwell™ culture system. White area is for Annexin V binding on DC culture in absence of cCD4+ G121. Results are representative of two independent experiments.
(TIF)
