# Supplementary figures and images for: Increased Synapse Formation Obtained by T Cell Epitopes Containing a CxxC Motif in Flanking Residues Convert CD4+ T Cells into Cytolytic Effectors
Source: PLoS One. 2012 Oct 9;7(10):e45366. doi: 10.1371/journal.pone.0045366 (PMC3467281; doi:10.1371/journal.pone.0045366)

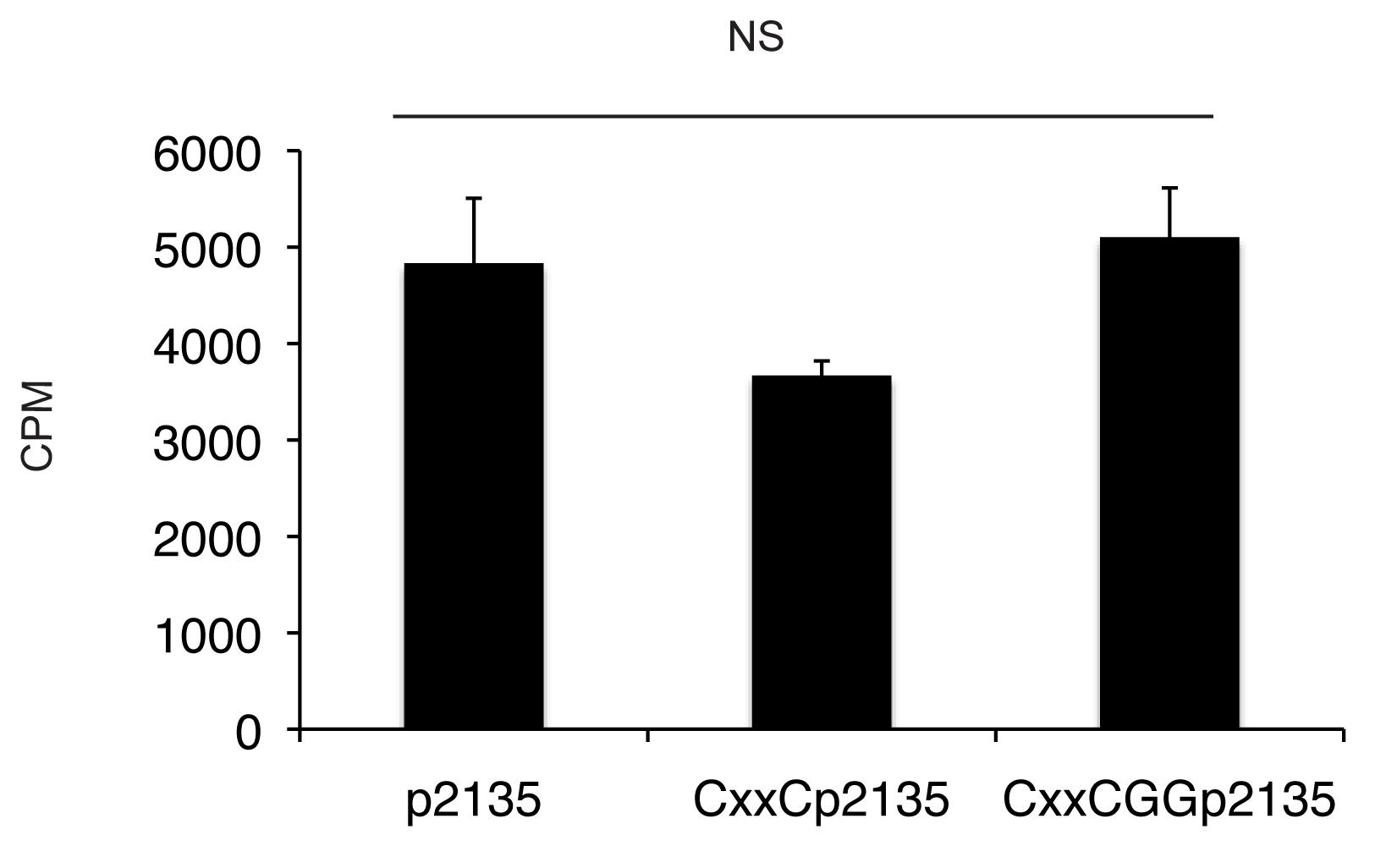

Supplement: Figure S1 — Effects of aminoacid residue substitution in the p21–35 flanking sequence. A p21–35 specific cytolytic CD4+ clone (G121) was cultured with T cell depleted mitomycin C-treated splenocytes in the presence of 0.1 µM of either natural sequence peptide p21–35 (CxxS motif), CxxCp2135 peptide (CxxC motif) or CxxCGGp21–35 (CxxCGG motif, with a linker made of two glycine residues). 3H-thymidine incorporation was measured after 72 h. Data representative of two experiments. Similar results were obtained with an alternative p21–35 specific cCD4+ clone R3TB7. Error bars represent 1 SD; One way Anova analysis of variance was done for comparing the three peptides (NS, p = 0.12). (TIF) [file pone.0045366.s001.tif]

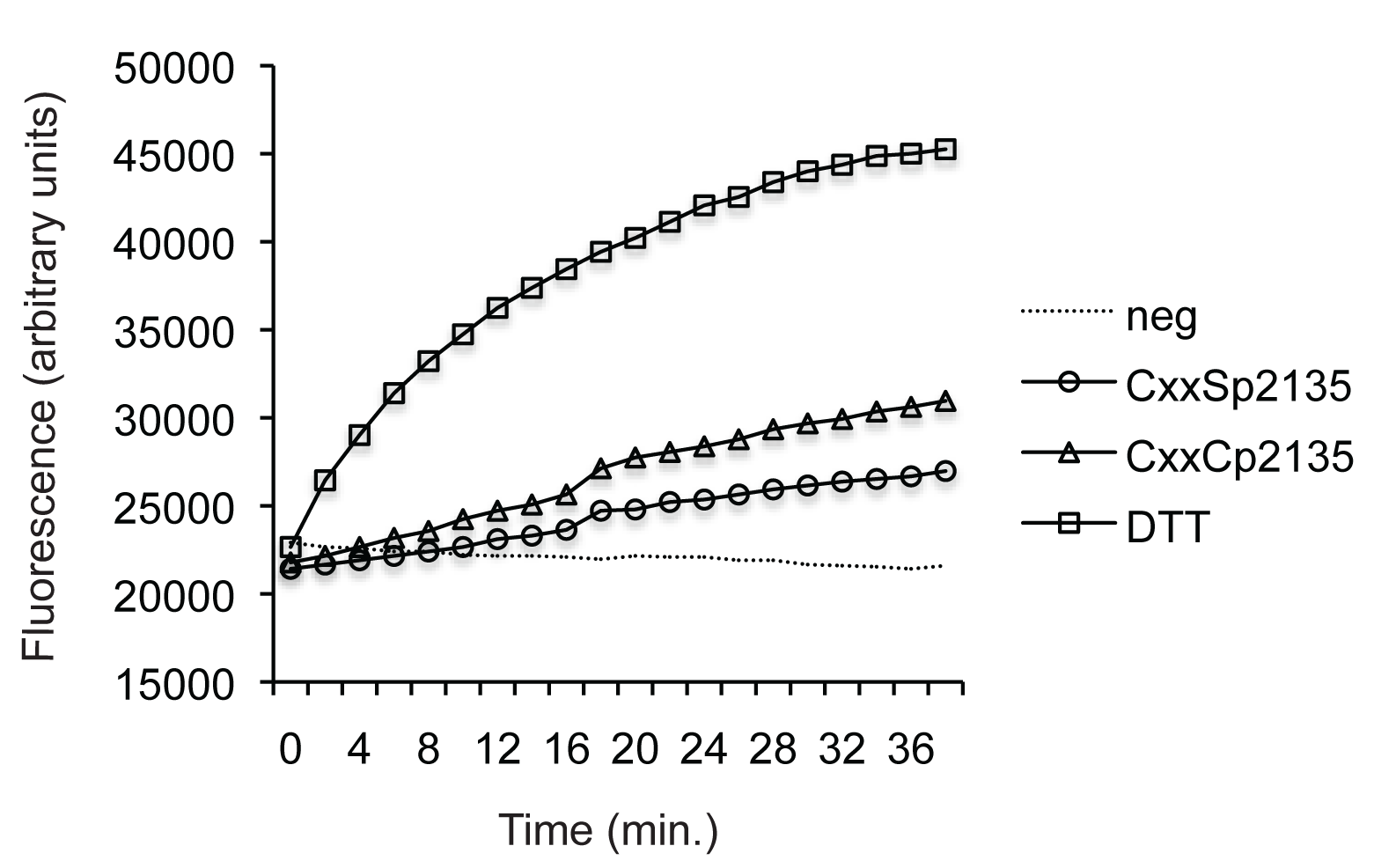

Supplement: Figure S2 — T cell epitopes containing a thioredoxin active motif reduce disulfide bridges in vitro. The redox capacity of peptides CxxSp21–35 or CxxCp21–35 (25 µM) was tested by time course experiment after incubation with self-quenched redox substrate (FITC-Gly-Cys-Asp)ox. The reaction was followed for 40 minutes after excitation at 495 nm and recording FITC emission at 530 mm. Dithiothreitol (DTT; 2 mM) was used as positive control. (TIF) [file pone.0045366.s002.tif]

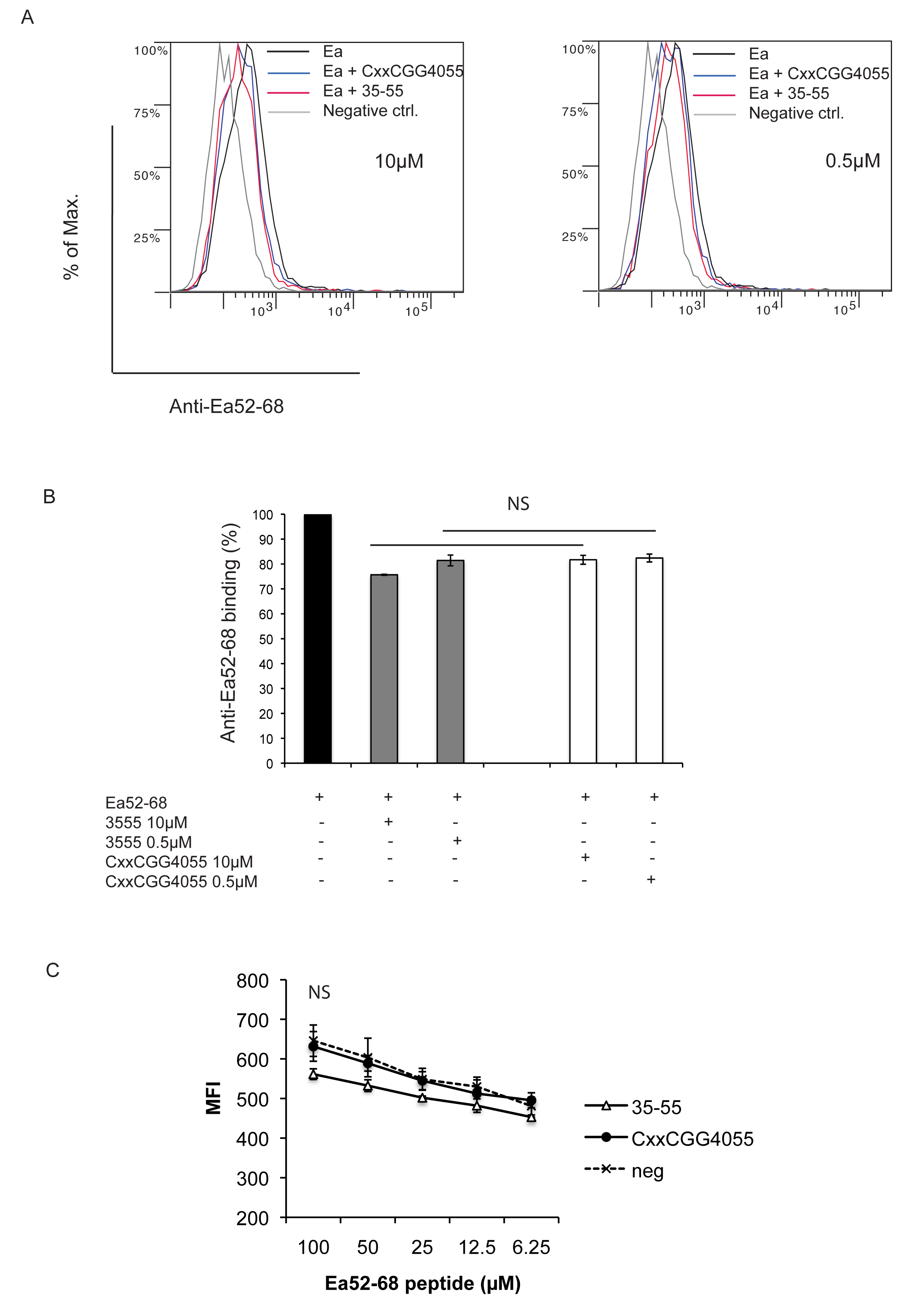

Supplement: Figure S3 — Insertion of a CxxC motif within flanking residues does not modify peptide affinity for MHC class II molecules. (A) Competition experiments for MHC class II binding were carried out by incubating B cells for 1 h at 37°C with Ea52–68 peptide (25 µM in each experiment), either alone (black histogram) or together with peptides MOG 35–55 (red histogram) or CxxCGG40–55 (blue histogram) at 10 µM (left panel) or 0.5 µM (right panel). After washing, binding of peptide Ea52–68 was detected with a fluorescent antibody (Y-Ae) recognizing the peptide Ea52–68/MHC class II complex. Left and right panel represent detection of the complex in the presence of 10 µM or 0.5 µM of competing peptides, respectively. (B) Decrease in signal was calculated from data obtained in (A). Non-parametric test (Mann-Whitney) showed no difference between peptides (p>0.3). Error bars show mean +/− SD. (C) B cells (5.104 cells/test) were loaded during 1 h with 10 µM of peptide MOG 35–55, 10 µM of peptide CxxCGG40–55, or kept unloaded (neg). After washing, cells were incubated with Ea52–68 peptide at different concentrations. After 1 h, cells were washed and peptide displacement was assessed by detecting Ea52–68/class II complex as in (A) (NS; p = 0.2) One-way anova test was used to compare the three curves. Error bars show mean +/− SD. (TIF) [file pone.0045366.s003.tif]

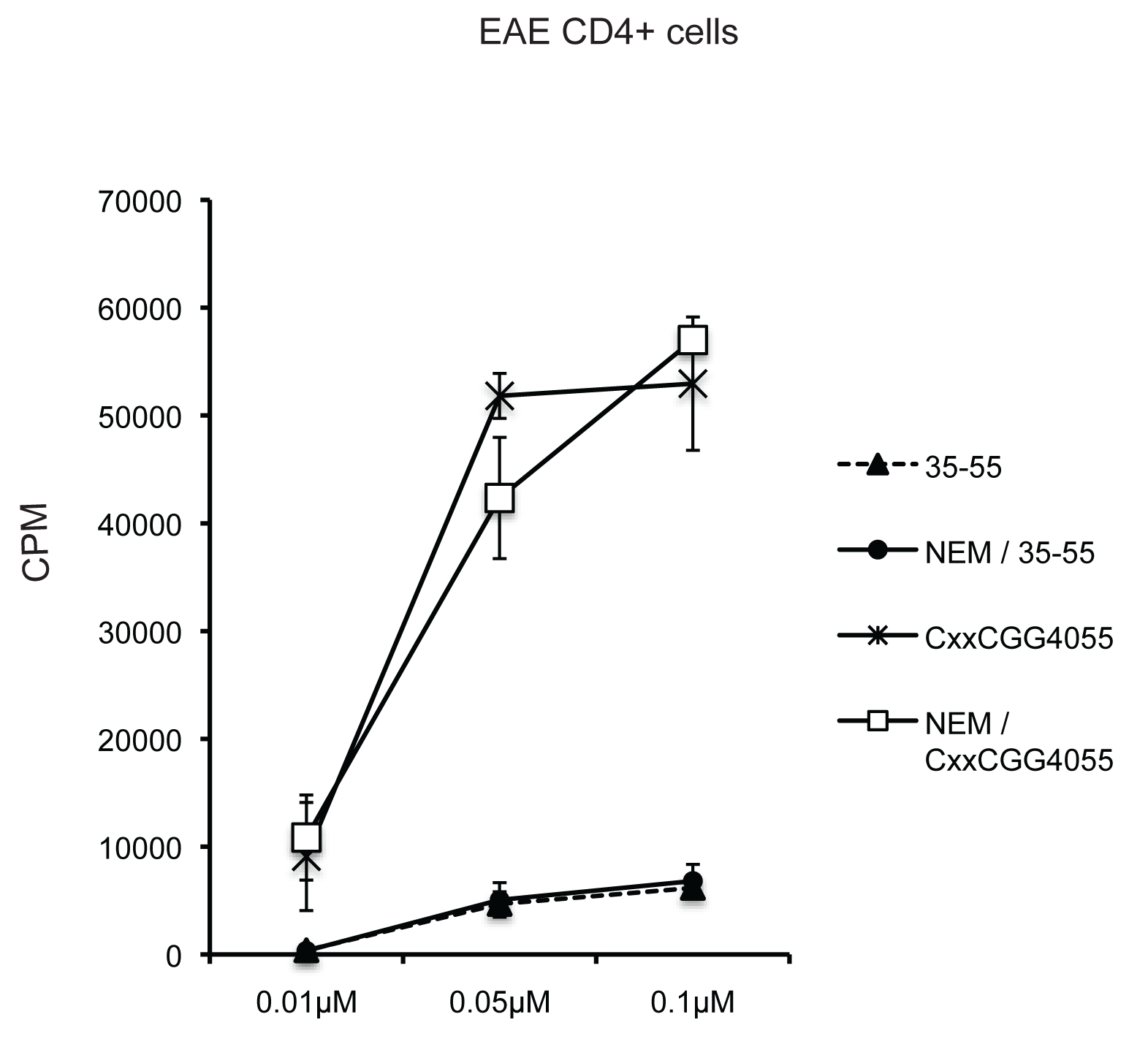

Supplement: Figure S4 — The effects resulting from inserting a CxxC motif do not depend on interaction with free surface thiols. A Th17 CD4 T cell line obtained from mice induced into EAE was stimulated with APC in the presence of MOG35–55 or CxxCGG40–55 peptides for 48 h. Where indicated, CD4 cells were pre-treated with 5 µM N-ethylmaleimide (NEM) before culture. 3H-thymidine was added for the last 12 h of culture. Error bars represent 1 SD. Data representative of two experiments. (TIF) [file pone.0045366.s004.tif]

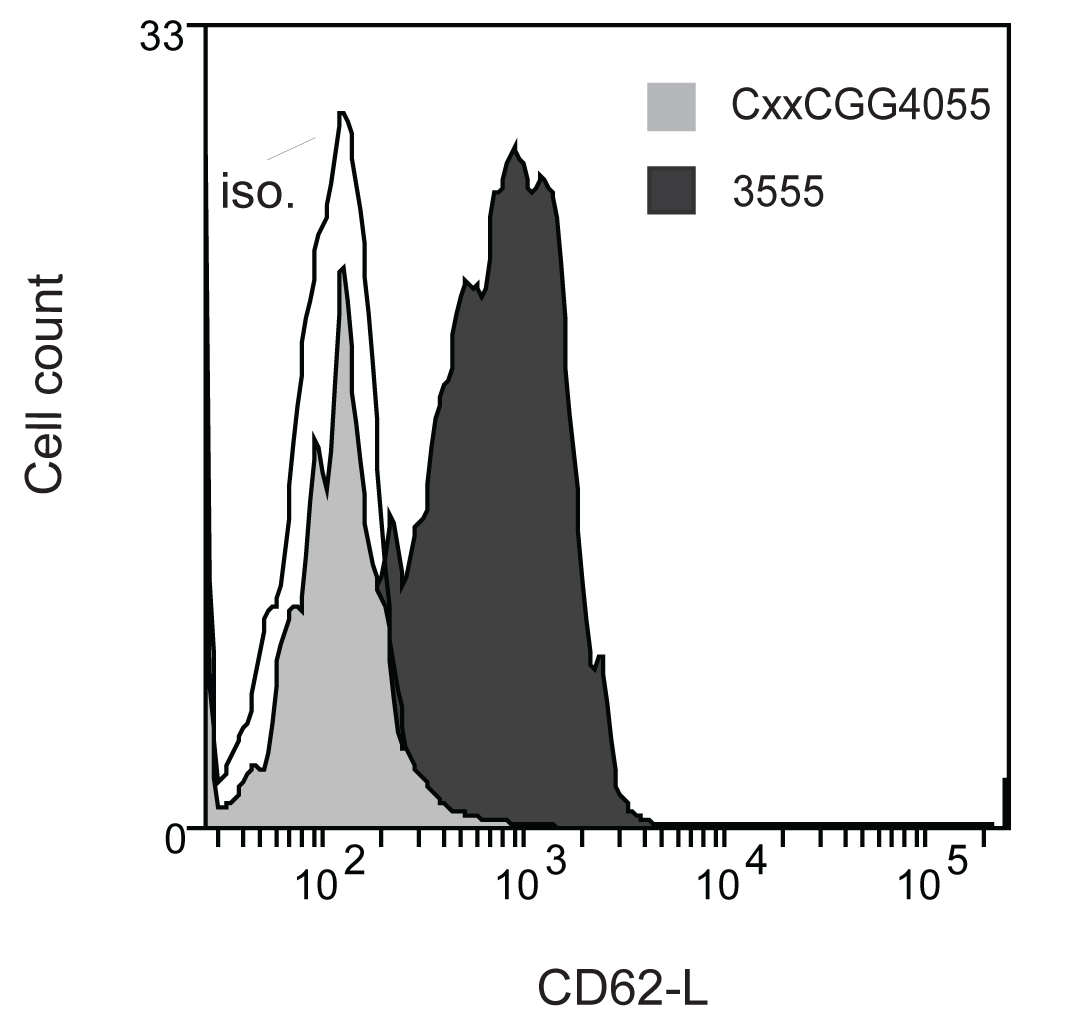

Supplement: Figure S5 — Increased maturation of naïve CD4+ T cells exposed to a CxxC-containing peptide. Naïve cells from 2D2 transgenic mice were polarized under Th17 conditions and analyzed for CD62L expression after a single stimulation (day 10) with 1 µM MOG CxxCGGp40–55 (grey histogram) or with 1 µM MOG 35–55 (black histogram). Open histogram is for isotype control antibody. Data representative of two experiments. (TIF) [file pone.0045366.s005.tif]

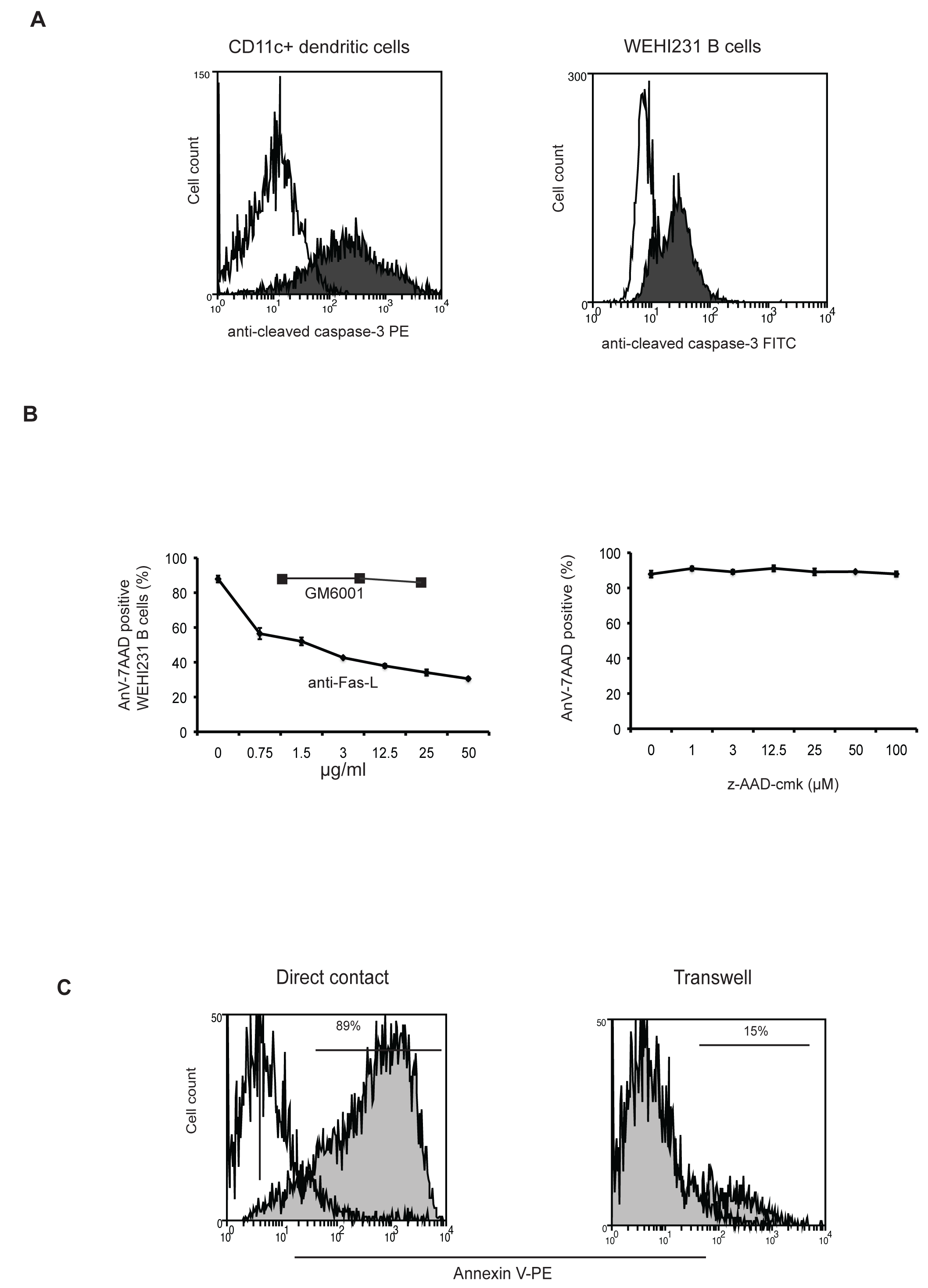

Supplement: Figure S6 — Induction of apoptosis in APC requires direct cell recognition and is mediated by Fas-L. (A) CD11c+ dendritic cells (DC) were obtained from BALB/c splenocytes by positive selection using magnetic beads and activated by LPS. DC (left panel) and WEHI-231 cells (right panel) were then loaded with 1 µM p21–35 peptide and co-cultured for 18 h with R3TB7 cCD4+ T cell (black areas). Open histograms represent anti-cleaved caspase-3 staining in DC or B cells cultured without T cells. Detection of cleaved caspase 3 expression was taken as a marker of cell apoptosis. Data is representative of a minimum of 3 independent experiments. (B) DiOC18 stained WEHI-231 B cells were loaded with 1 µM p21–35 peptide and incubated with R3TB7 (1/1 ratio) in the presence of increasing concentrations of anti-FasL antibody or of the metalloprotease inhibitor GM6001, as indicated (left panel). Increasing concentrations of an antagonistic peptide of GZ-B (Z-AAD-fmk) were also tested (right panel). Apoptosis of WEHI-231 cells was measured after 18 hours and staining with Annexin V and 7AAD. Error bars represent 1 SD. Data representative of two experiments. (C) CD11c+ DC were loaded with p21–35 peptide and incubated in the presence of cCD4+ G121 T cell clone for 18 h at a 1/1 ratio. The left panel shows DC apoptosis measured by Annexin V binding (gated on Vβ8 negative cells). The right panel shows the same experiment but with peptide-loaded DC and cCD4+ G121 cells separated by a semi-permeable membrane in a Transwell™ culture system. White area is for Annexin V binding on DC culture in absence of cCD4+ G121. Results are representative of two independent experiments. (TIF) [file pone.0045366.s006.tif]
